# NDUFS3 promotes proliferation via glucose metabolism reprogramming inducing AMPK phosphorylating PRPS1 to increase the purine nucleotide synthesis in melanoma

**DOI:** 10.1038/s41418-025-01525-4

**Published:** 2025-05-22

**Authors:** Guohang Xiong, Fang Yun, Lu Jiang, Zihan Yi, Xiaojia Yi, Lijuan Yang, Xuedan Zhang, Xiaoyu Li, Zhe Yang, Qiao Zhang, Buqing Sai, Yingmin Kuang, Yuechun Zhu

**Affiliations:** 1 Department of Biochemistry and Molecular Biology, School of Basic Medical Sciences, Kunming Medical University, Kunming, 650500 China; 2 Research Center for Clinical Medicine, First Affiliated Hospital of Kunming Medical University, Kunming Medical University, Kunming, 650032 China; 3 Department of Pathology, The First Affiliated Hospital of Nanjing Medical University (Jiangsu Province Hospital), Nanjing, 210000 China; 4 Department of Medical Oncology, The Third Affiliated Hospital of Kunming Medical University (Tumor Hospital of Yunnan Province), Kunming, 650118 China; 5 Department of Pathology, The Second Affiliated Hospital of Kunming Medical University, Kunming, 434000 China; 6 Department of Pathology, The First Affiliated Hospital of Kunming Medical University, Kunming, Yunnan Province 650032 China; 7 Department of Organ Transplantation, The First Affiliated Hospital of Kunming Medical University, Kunming, 650032 China

**Keywords:** Cancer metabolism, Prognostic markers, Cancer metabolism

## Abstract

NADH dehydrogenase [ubiquinone] iron-sulfur protein 3 (NDUFS3) is the core subunit of the respiratory chain complex I (CI). We found NDUFS3 were abnormally elevated in human melanoma and promoted melanoma proliferation. Furthermore, NDUFS3 could promote the oxidative phosphorylation (OXPHOS) and the pentose phosphate pathway (PPP), as well as attenuated glycolysis. As NDUFS3-mediated the metabolic changes of OXPHOS and glucose metabolism, melanoma cells produced more ATP, resulting in the inhibition of AMP kinase (AMPK). AMPK induced phosphoribosyl pyrophosphate synthetase1 (PRPS1) phosphorylation, which resulted in suppressed PRPS1 activity. Briefly, the NDUFS3-AMPK-PRPS1 signaling axis coupled OXPHOS, glucose metabolism, and purine nucleotide biosynthesis to regulate melanoma proliferation. Our study highlighted an unrecognized role for NDUFS3 in melanoma, which might be used as a potential therapeutic target for the treatment of this type of cancer.

**Figure:**
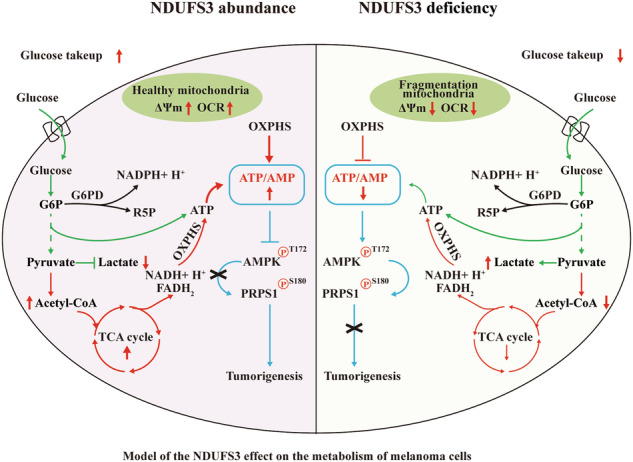
NDUFS3 regulating PRPS1 activity through AMPK to affect melanoma proliferation.

NDUFS3 regulating PRPS1 activity through AMPK to affect melanoma proliferation.

## Introduction

Previously, it was believed that due to the impairment of mitochondrial function in cancer cells, the process of oxidative phosphorylation (Oxidative phosphorylation system, OXPHOS) was inhibited, causing cancer cells to mainly generate ATP through aerobic glycolysis, which is known as the Warburg effect [1, 2]. However, an increasing number of studies have shown that the mitochondria of tumor cells can undergo metabolic reprogramming to meet the high energy demands during the proliferation and metastasis of tumor cells. In prostate cancer [3], leukemia [4, 5], lymphoma [6], and pancreatic ductal adenocarcinoma [7], OXPHOS is upregulated. Approximately 35–50% of BRAF-mutant and wild-type melanoma cell lines as well as melanoma patients exhibit high oxidative phosphorylation activity [8]. The metastasis of melanoma depends on oxidative phosphorylation [9, 10]. Increased levels of OXPHOS contribute to maintaining the stemness of melanoma [11]. Breast cancer [12], prostate cancer [13], human glioma [14], and melanoma [15], can exhibit the phenomenon of glycolysis and OXPHOS metabolic symbiosis. When lactate dehydrogenase A and lactate dehydrogenase B in melanoma cells B16-F10 are knocked out simultaneously, the OXPHOS of the cells increases significantly, and it can even become completely independent of glycolysis [16]. Therefore, effectively targeting the heterogeneity of tumor glycolysis and OXPHOS metabolism can serve as a new strategy for melanoma treatment.

Mitochondria are organelles responsible for bioenergy and biosynthesis. They play a crucial role in regulating tumor energy and material metabolism, including the tricarboxylic acid cycle (TCA cycle) and electron transfer respiratory chain/OXPHOS. This regulation leads to the production of ATP, nicotinamide adenine dinucleotide phosphate (NADPH), and raw materials for synthesizing amino acids, lipids, and nucleotides [17, 18]. Complex I is the first and largest enzyme in the respiratory chain and one of the entry points of electrons utilized in the OXPHOS system [19, 20]. Studies have shown that the protein expression level of NDUFS3, the core subunit of mitochondrial complex I (CI), is positively correlated with the activity of mitochondrial complex I [21–23]. Low expression of NDUFS3 inhibits the function of the respiratory chain, resulting in lymphoma cell death [24]. In addition, knockdown of NDUFS3 expression promotes aerobic glycolysis in breast cancer [25] and renal carcinoma [26]. However, the role of NDUFS3 in human melanoma development is unclear.

Here, we confirmed that NDUFS3 plays a key role in regulating glucose metabolism, OXPHOS, and purine nucleotide metabolism in melanoma. NDUFS3 activated the aerobic oxidation of glucose and OXPHOS and downregulated glycolysis, thereby promoting the production of greater amounts of ATP and inhibiting AMPK activity in melanoma. AMPK modulates the activity of phosphoribosyl pyrophosphate synthetase 1 (PRPS1), which is the first rate-limiting enzyme in purine nucleotide biosynthesis. When AMPK was activated, the activity of PRPS1 was antagonized, thus leading to a marked decrease in purine nucleotide synthesis in melanoma cells. In brief, we confirmed that NDUFS3 was tightly linked to melanoma proliferation by regulating the NDUFS3-AMPK-RPPS1 signaling axis.

## Results

### NDUFS3 expression is strongly increased in melanoma and promotes the proliferation of melanoma cells

**Fig. 1:**
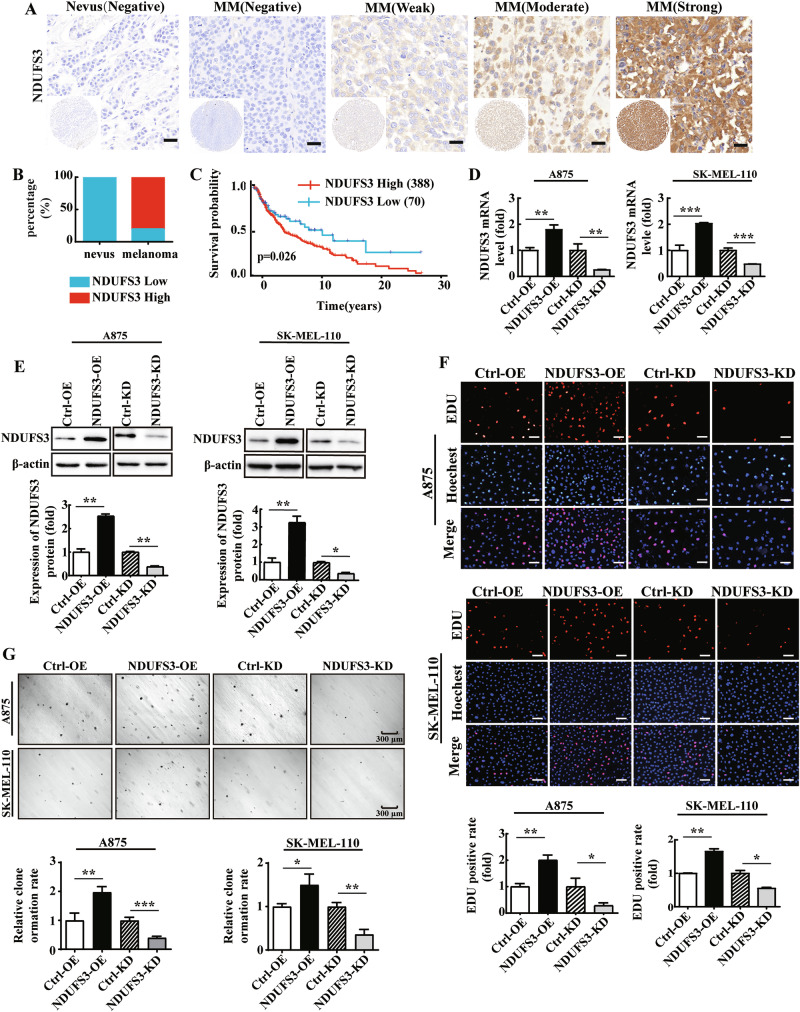
NDUSF3 is highly expressed in melanoma cells and promotes their proliferation. **A** The expression of NDUFS3 in nevi and melanoma was detected. Optical microscopy with a 40× objective lens was used to analyze the IHC results. Scale bars=50 μm. **B** Based on the IHC staining score results, the number of Ki67-low (4/89) and high-Ki67 (85/89) cases in melanoma and Ki67-low (2/10) and high-Ki67 (8/10) cases in nevi was counted and expressed as percentages. **C** Using the TCGA database and Kaplan Meier survival analysis method, we evaluated the correlation between NDUFS3 expression levels and survival prognosis in melanoma patients. A875 and SK-MEL-110 cells with stable NDUFS3 knockdown (hereinafter abbreviated as KD) or overexpression (hereinafter abbreviated as OE) were established. The mRNA and protein expression of NDUSF3 were measured by qPCR **D** and Western blotting (**E**). **F** EdU was used to evaluate the proliferation of NDUFS3-overexpressing or NDUFS3-knockdown melanoma cells. Scale bars = 20 μm. **G** The soft agar colony formation assay was used to determine the cell proliferation ability after NDUFS3 overexpression and knockdown in A875 and SK-MEL-110 cells. The data represent three independent experiments, each carried out in triplicate. The data are presented as the means ± SD and were analyzed by Student’s t test. *p < 0.05; **p < 0.01; ***p < 0.001.

To investigate the relationship between the expression of NDUFS3 and the proliferation of melanoma, as well as the clinical diagnosis of melanoma. A melanoma tissue microarray was used to detect NDUFS3 levels by immunohistochemistry (IHC). The detailed characteristics of the melanoma patients are listed in Table S1. The tissue specimens included 10 nevi, 76 malignant melanomas (MM) and 14 metastatic melanomas (MM) (for which MM tissue was unavailable). Typical images are shown in Fig. 1A. As summarized in Table 1 and Fig. 1B, no positive signals were detected in the nevi (0/10), whereas NDUFS3 expression was strongly increased in melanoma.We also calculated the expression levels of Ki67 in the same tissue chip. Upon conducting Pearson correlation analysis, we identified a significant positive correlation between the expression levels of NDUFS3 and the Ki67 in melanoma tissues (*r* = 0.67, *p* < 0.001). This finding suggests that high expression of NDUFS3 is indeed closely associated with the proliferative activity of melanoma cells (Fig. S1). In addition, the clinical data of 458 patients with melanoma were derived from the TCGA database. We found that higher levels of NDUFS3 were associated with a lower survival rate (Fig. 1C).

**Table 1:** Immunohistochemistry of NDUFS3 and Ki67 and PRPS1 and p-PRPS1(180) expression in melanoma.

Antibody	Tissue type	No. samples	Staining			
			Negative	Weak	Moderate	Strong
NDUFS3	Nevus	10	10	0	0	0
	melanoma	89	8	10	29	47
Ki67	Nevus	10	2	0	7	1
	melanoma	89	1	3	43	42
PRPS1	Nevus	10	0	5	5	0
	melanoma	89	0	11	38	40
p-PRPS1(180)	Nevus	10	1	0	1	8
	melanoma	89	8	58	21	2

Next, human melanoma cells A875 and SK-MEL-110 were infected with lentivirus to obtain melanoma cell lines with stable NDUFS3 overexpression (hereinafter abbreviated as NDUFS3 OE) or knockdown (hereinafter abbreviated as NDUFS3 KD). Western blotting revealed that the greatest interference efficiency of NDUFS3 occurred in LV-NDUFS3-RNAi-2 cells (Fig. S2), which were used as a stable cell line for NDUFS3 knockdown. Overall, these data indicated that both NDUFS3 overexpression and knockdown cell lines were successfully established (Fig. 1D–E). We further explored the effects of NDUFS3 on melanoma cell proliferation in vitro via Edu assay and colony forming ability. The percentage of NDUFS3-overexpressing cells marked with red fluorescence was greater than that of control cells, while NDUFS3 knockdown cells were the opposite (Fig. 1F). After overexpressing NDUFS3 in A875 and SK-MEL-110 cells, the number of cell colony formations increased. In contrast, in A875 and SK-MEL-110 cells with NDUFS3 knockdown, the number of cell colony formations decreased (Fig. 1G). In addition, Flow cytometry and western blot analyses confirmed that NDUFS3 can regulate the expression of cell cycle proteins in melanoma cells, increase the proportion of cells in the S phase, accelerate cell division, and thus promote the proliferation of melanoma cells (Fig. S3A–C). These results suggest that NDUFS3 is highly expressed in human melanoma tissues and is negatively associated with patient survival and prognosis, as well as promoting the proliferation of melanoma cells.

### NDUSF3 promotes melanoma development in vivo

**Fig. 2:**
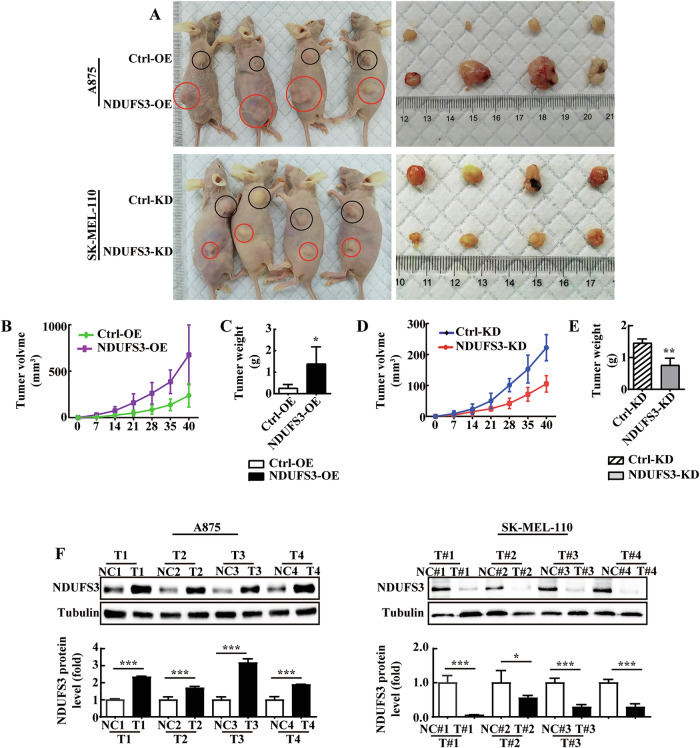
NDUFS3 promotes melanoma proliferation in vivo. BALB/c nude mice were injected with stably transfected A875 or SK-MEL-110 melanoma cells and the corresponding controls. Representative images of mice with control (top) and NDUFS3-overexpressing/NDUFS3-knockdown (lower) xenograft tumors **A**. The tumor volume (**B, D**) and body weight (**C, E**) were measured. **F** NDUFS3 protein levels in tumors from each group were measured by western blot analysis. The tumor volume data were statistically analyzed by two-way ANOVA. The other data, representing three independent experiments each performed in triplicate, were analyzed using the unpaired Student’s t-test. *P < 0.05, **P < 0.01, ***P < 0.001.

We found that melanoma tumor growth was significantly promoted in mice inoculated with NUDFS3-overexpressing cell (Fig. 2A–C), while melanoma tumor growth was inhibited in mice bearing stable NDUFS3-knockdown cell xenografts (Fig. 2A, D, E). We further compared the expression of NDUFS3 in the tumor tissues of each group. Our results indicated that NDUFS3 levels in the tumors were positively correlated with the melanoma tumor volumes in each group (Fig. 2F). These results suggested that NDUFS3 could promote the growth of melanoma in vivo.

### NDUSF3 affects mitochondrial morphology and promotes OXPHOS

**Fig. 3:**
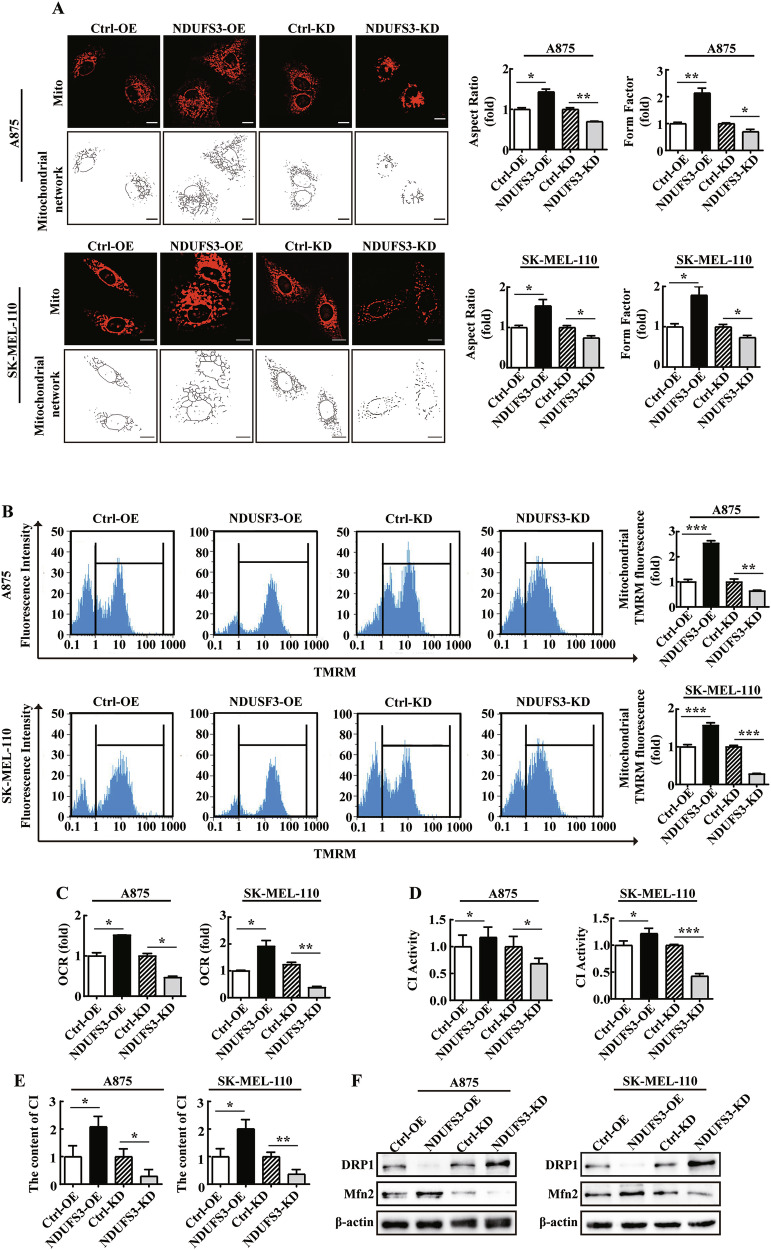
NDUSF3 impacts mitochondrial morphology and function. **A** Representative images of the effect of NDUFS3 overexpression or knockdown on mitochondrial phenotypes in A875 and SK-MEL-110 cells, as determined by immunofluorescence staining. Mitochondria were labeled with Red FP Mitochondrion (red). Images were acquired with a 100× oil immersion objective. Scale bars =10 μm. The aspect ratio and form factor were used as the standards for assessing mitochondrial network complexity (the number of cells ranged from 80 to 100). **B** Flow cytometry analysis was used to evaluate the relative changes in mitochondrial TMRM fluorescence in the stable A875 and SK-MEL-110 cell lines and the controls. **C** The oxygen consumption rate (OCR) of melanoma cells with NDUFS3 overexpression and knockdown was determined by using fluorescence spectrophotometer. **D** Mitochondrial complex I (CI) activity of A875 and SK-MEL-110 cells with NDUFS3 overexpression or knockdown was detected by spectrophotometry at 450 nm wavelength. **E** ELISA analysis was used to analyze the effect of overexpression or knockdown of NDUFS3 on the content of CI in melanoma cells. **F** Western Blot detection of Mfn2 and DRP1 expression in NDUFS3 overexpression or knockdown melanoma cell lines. The data are presented as the means ± SD. These data represent three independent experiments, each carried out in triplicate. *p < 0.05, **p < 0.01, and ***p < 0.001. *p* values were calculated by Student’s t test.

Therefore, we investigated the effects of NDUFS3 on mitochondrial morphology and dynamics in melanoma cells. The aspect ratio and form factor were used as two parameters involved in the assessment of mitochondrial morphology [27]. The aspect ratio serves as an index for evaluating mitochondrial length, while the form factor is a metric that considers both the length and branching degree of mitochondria [27, 28]. In the mitochondrial quantitative images, the melanoma cells stably transfected with NDUFS3 were marked with Deep Red mitochondrion (Mito) (Fig. 3A, top panel), and morphometric quantitative measurements were acquired by Image J (Fig. 3A, bottom panel). The results indicated that the complexity of the mitochondrial network increased in both the NDUFS3-overexpressing A875 and SK-MEL-100 cells. However, NDUFS3-knockdown melanoma cells exhibited reduced mitochondrial complexity (Fig. 3A).

To evaluate the role of NDUFS3 in mitochondrial dynamics, the mitochondrial membrane potential (ΔΨ_mt_) and the oxygen consumption rate (OCR) were measured. We found that the ΔΨ_mt_ was significantly increased in NDUFS3-overexpressing A875 and SK-MEL-110 cells. In contrast, the ΔΨ_mt_ was lower in NDUFS3-knockdown A875 and SK-MEL-110 cells than in control cells (Fig. 3B). The overexpression of NDUFS3 increases the OCR, CI enzyme activity and content in melanoma cells, while NDUFS3 knockdown decreases these parameters (Fig. 3C–E). Additionally, NDUFS3 overexpression in melanoma cells leads to increased Mfn2 and decreased DRP1, with the opposite effects observed upon NDUFS3 knockdown (Fig. 3F).

These findings suggested that NDUFS3 overexpression enhanced mitochondrial complexity and promoted OXPHOS and that NDUSF3 deficiency induced mitochondrial fragmentation and mitochondrial dysfunction in melanoma cells.

### NDUSF3 promotes the aerobic oxidation of glucose and attenuates glycolysis in melanoma cells

**Fig. 4:**
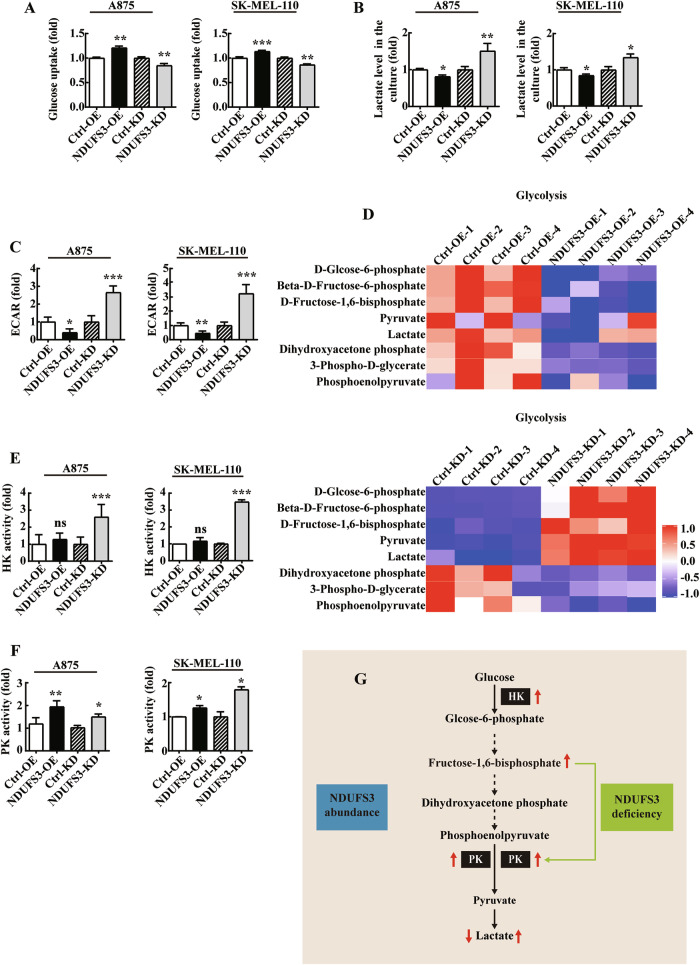
The effects of NDUFS3 on glucose metabolism in melanoma cells. Glucose uptake (**A**) and lactate production (**B**) in NDUFS3-knockdown or NDUFS3-overexpressing A875 (A and B, left) and SK-MEL-110 (A and B, right) cells. **C** The effect of NDUFS3 on the ECAR in A875 (left) and SK-MEL-100 (right) cells with stable knockdown or overexpression of NDUFS3. The ECAR results were obtained by using a BBcellProbep61 probe. **D** Heatmap analysis showing the changes in metabolite sets in NDUFS3-knockdown and NDUFS3-overexpressing SK-MEL-110 cells and controls (*n* = 4). **E, F** The activity of hexokinase (HK) **F** and pyruvate kinase (PK) **F** in NDUFS3-deficient or NDUFS3-overexpressing A875 and SK-MEL-110 cells and the control cells. **G** Schematic of the glycolysis (black arrows) pathway. The enzymes are highlighted in white. The activity of the data is presented as the mean ± SD. These data represent three independent experiments, each carried out in triplicate. *p* values were calculated by Student’s t test,*p < 0.05, **p < 0.01, ***p < 0.001.

The Warburg effect suggests that cancer cell with increased glucose uptake and lactate release can maintain rapid proliferation. When the oxygen supply is sufficient, tumor cells obtain energy via glycolysis and OXPHOS [29–32]. The exact mechanism by which NDUFS3-induced alterations in OXPHOS affect glucose metabolism in melanoma cells remains unknown. Firstly, we found that the proliferation of melanoma cells overexpressing NDUFS3 caused an increase in glucose consumption, while it downregulated the levels of lactate in the culture supernatant and the extracellular acidification rate (ECAR). In contrast, NDUFS3-knockdown melanoma cells showed opposite trends (Fig. 4A–C).

To further determine the global effect of NDUFS3 on the metabolism of melanoma, targeted metabolomics profiling was performed in stable NDUFS3-overexpressing or knockdown SK-MEL-110 cells. A total of 30 major metabolites derived from the processes of glycolysis, the TCA cycle and PPP metabolism were analyzed by liquid chromatography-tandem mass spectrometry (LC‒MS/MS). The results showed that the levels of key intermediates involved in glycolysis were significantly decreased following the overexpression of NDUFS3 in SK-MEL-110 cells (Fig. 4D). In addition, the knockdown of NDUFS3 increased the levels of key intermediates involved in glycolysis, including both pyruvate and lactate (Fig. 4D). Moreover, we found two interesting phenomena. First, the increase or decrease in glucose uptake in NDUFS3-overexpressing or NDUFS3-knockdown cells was inconsistent with the increase or decrease in glucose-6-phosphate content. To answer this question, the activity of hexokinase (HK), which is the first glycolytic enzyme, was examined. HK activity was elevated in NDUFS3-knockdown A875 and SK-MEL-110 melanoma cells, whereas there is no significant difference between NDUFS3-overexpressing cells and control cells (Fig. 4E). Similar results on the role of HK in the regulation of glycolysis by NDUFS3 have been previously reported [25, 26]. In addition, the levels of three metabolites (3-phospho-D-glycerate, phosphoenolpyruvate and dihydroxyacetone-phosphate), which represent glycolysis, were not increased in NDUFS3-knockdown cells (Fig. 4D). We found that pyruvate kinase (PK) activity was greater in NDUFS3-knockdown A875 and SK-MEL-110 cells than in the corresponding control cells (Fig. 4F). However, after the overexpression of NDUFS3, the PK enzyme activity in melanoma cells is also stronger than that in the control cells(Fig. 4F). D-fructose-1,6-bisphosphate can activate PK, which accelerates the conversion of phosphoenolpyruvate to pyruvate [33]. Interestingly, the PK activity of the NDUFS3-overexpressing cells was also greater than that of the control cells, possibly because of a compensatory mechanism to produce higher levels of pyruvate for the TCA cycle.

These results suggested that NDUSF3 promotes aerobic oxidative metabolism and inhibits glycolysis in melanoma cells through positive and negative feedback regulation of HK and PK enzyme activities (Fig. 4G).

### NDUFS3 promotes the TCA cycle in melanoma cells

**Fig. 5:**
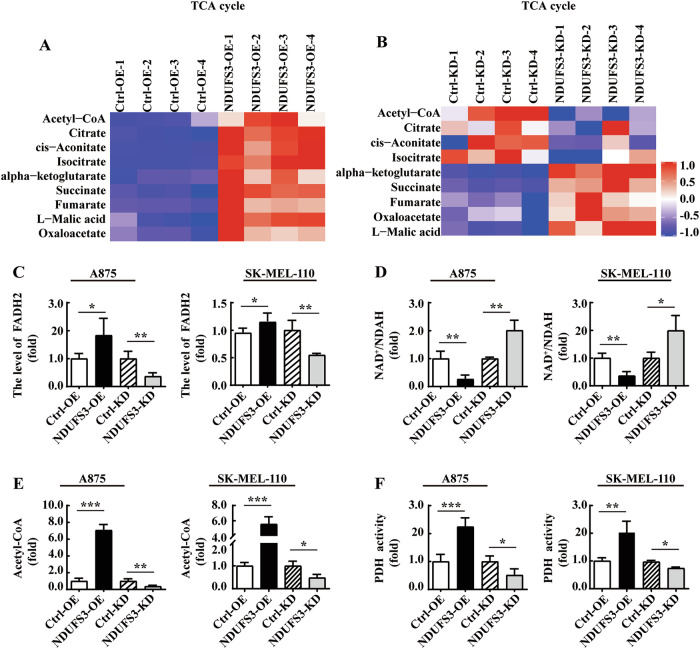
The effects of NDUFS3 on the TCA cycle in melanoma cells. **A** Heatmaps of metabolites involved in the TCA cycle in SK-MEL-110 cells with NDUFS3 knockdown or overexpression. **B** The relative pyruvate dehydrogenase (PDH) activity in stably transfected SK-MEL-110 cells. The levels of FADH2 (**C**), NAD^+^/NADH (**D**), Acetyl-CoA (**E**), and pyruvate dehydrogenase (PDH) activity (**F**) in NDUFS3-knockdown and NDUFS3-overexpressing A875 and SK-MEL-110 cell lines and the control. **G** Schematic of the glycolysis (black arrows) and TCA cycle (blue or green) pathways. The enzymes are highlighted in white. The activity of the data is presented as the mean ± SD. These data represent three independent experiments, each carried out in triplicate. *p* values were calculated by Student’s t test, *p < 0.05, **p < 0.01, ***p < 0.001, ns indicates no significant difference.

An important function of the TCA cycle is to provide flavin adenine dinucleotide (FADH2) and NADH for OXPHOS. The LC‒MS/MS results also showed that overexpression of NDUFS3 promoted the TCA cycle in SK-MEL-110 melanoma cells, with intermediate products such as Acetyl-CoA, isocitrate, α-ketoglutarate (Fig. 5A); On the contrary, knocking down SK-MEL-110 NDUFS3 resulted in a decrease in Acetyl-CoA, citric acid, and isocitrate content (Fig. 5B). Similarly, we have examined the levels of FADH2, the NAD^+^/NADH ratio, and Acetyl-CoA in A875 cells with overexpression or knockdown of NDUFS3. The results show that, consistent with the abnormal expression of NDUFS3 in SK-MEL-110 cells, overexpression of NDUFS3 leads to an increase in FADH2, a decrease in the NAD^+^/NADH ratio, and an elevation in Acetyl-CoA levels in A875 cells. Conversely, knockdown of NDUFS3 results in the opposite effects on FADH2, the NAD^+^/NADH ratio, and Acetyl-CoA levels in A875 cells (Fig. 5C–E). In addition, compared with the control group cells, the activity of pyruvate dehydrogenase (PDH), which catalyzes the production of Acetyl-CoA from pyruvate, was upregulated in A875 and SK-MEL-110 melanoma cells overexpressing NDUFS3, while knocking down NDUFS3 resulted in downregulation of PDH activity (Fig. 5F).

Although LC‒MS/MS results showed that knocking down NDUFS3 inhibited the TCA cycle in melanoma cells, it is worth noting that in SK-MEL-110 cells knocked down by NDUFS3, the content of five metabolites in the TCA cycle reaction from α-ketoglutarate to malic acid was upregulated (Fig. 5B). This may be a self-compensatory mechanism of tumor cells supplementing α-ketoglutarate with glutamine [34, 35], aimed at compensating for the reduced ATP production caused by mitochondrial OXPHOS dysfunction caused by knocking down NDUFS3.

Taken together, these results indicated that the overexpression of NDUFS3 may not result in the excretion of glucose metabolites such as lactate, which tends to produce additional ATP and OXPHOS fuel by improving the efficiency of aerobic oxidative metabolism and the TCA cycle. However, following the knockdown of NDUFS3 expression, the cells were more likely to produce ATP via glycolysis.

### NDUFS3 promotes the PPP and purine nucleotide biosynthesis in melanoma cells

**Fig. 6:**
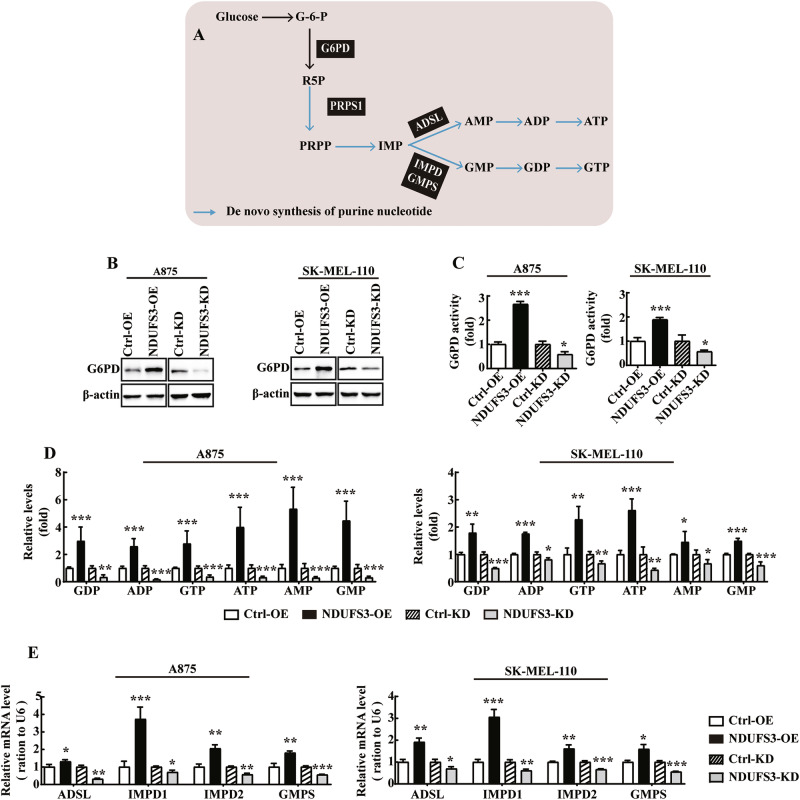
The PPP and purine nucleotide metabolism are inhibited in NDUFS3-knockdown melanoma cells. **A** The PPP (black) and de novo synthesis of purine nucleotides (blue) and deoxy pyrimidine nucleotide (red) metabolic pathways. The enzymes are highlighted in red. Glucose-6-phosphate dehydrogenase (G6PD), pyrophosphate synthetase 1 (PRPS1), adenyl succinate lyase (ADSL), GMP synthase (GMPS), IMP dehydrogenase 1/2 (IMPD1/2). G6PD enzymatic activity (**B**) and G6PD protein levels (**C**) were measured in NDUFS3-knockdown and NDUFS3-overexpressing A875 and SK-MEL-110 cells. **D** The abundance of nucleotide involved in nucleotide biosynthesis metabolism in A875 and SK-MEL-110 overexpressing cells and control cells. **E** qPCR analysis of the expression of genes encoding enzymes involved in nucleotide biosynthesis in A875 (left) and SK-MEL-110 (right) cells with stable knockdown or overexpression of NDUFS3. *p* values were calculated by using Student’s *t* test. *p < 0.05; **p < 0.01; ***p < 0.001.

As a branch of glucose metabolism, the main function of the Pentose Phosphate Pathway (PPP) is to provide ribose 5-phosphate (R5P) for nucleotide synthesis [36]. To further explore the roles of NDUFS3 in the PPP and purine nucleotide metabolism (Fig. 6A). Initially, the amount and activity of the key PPP enzyme glucose-6-phosphate dehydrogenase (G6PD) were examined. Overexpression of NDUFS3 increased G6PD activity and G6PD protein levels, whereas knockdown of NDUFS3 decreased G6PD and G6PD protein levels in both the A875 and SK-MEL-110 cell lines (Fig. 6B, C). In addition, following the A875 and SK-MEL-110 cell overexpression or knockdown of NDUFS3, the levels of nucleoside triphosphates (NTPs), such as GTP and ATP, and of nucleoside diphosphates (NDPs), such as GDP and ADP, were upregulated or downregulated, respectively (Fig. 6D). NDUFS3-overexpressing cells produced more AMP and GMP than did the control cells, whereas the NDUFS3-knockdown cells produced less AMP and GMP than did the control cells (Fig. 6D).

Moreover, RT‒PCR analysis revealed that the abundance of key enzymes involved in purine nucleotide metabolism, such as adenyl succinate lyase (ADSL), IMP dehydrogenase 1/2 (IMPD1/2), and GMP synthase (GMPS), was significantly decreased in NDUFS3-knockdown melanoma cells (Fig. 6E). However, the levels of key enzymes involved in purine nucleotide metabolism were increased in NDUFS3-overexpressing melanoma cells (Fig. 6E). We also measured the levels of Uridine Diphosphate (UDP), Uridine Triphosphate (UTP) and Cytidine Triphosphate (CTP) in A875 and 110 cells with NDUFS3 knockdown or overexpression and found that compared to control cells, there was no significant difference in the levels of UDP, UTP, and CTP in melanoma cells with abnormal NDUFS3 expression (Fig. S4).

An additional central role of the PPP is to maintain cellular redox homeostasis by producing NADPH and glutathione (GSH) [36, 37]. The results indicated that NDUFS3-overexpressing melanoma cells exhibited an increase in the NADPH/NADP^+^ ratio (Fig. 5SA). Conversely, a marked decrease in the NADPH/NADP^+^ ratio was noted following the knockdown of NDUFS3 expression in A875 and SK-MEL-110 cells (Fig. 5SA). The overexpression of NDUFS3 increased the GSH/GSSG ratio in A875 and SK-MEL-110 cells. In contrast, knockdown of NDUFS3 expression resulted in a decrease in the GSH/GSSG ratio (Fig. 5SB). Taken together, the results of the present study indicated that NDUFS3 promoted melanoma cell proliferation by upregulating the de novo synthesis of purine nucleotides (Fig. 5SC).

### PRPS1 phosphorylation is positively correlated with NDUFS3 expression in melanoma

To further examine the mechanism by which NDUFS3 promotes purine nucleotide synthesis, we focused on the first enzyme of de novo nucleotide synthesis, PRPS1, and p-PRPS1(S180) was in its low or inactive state [38, 39].

**Fig. 7:**
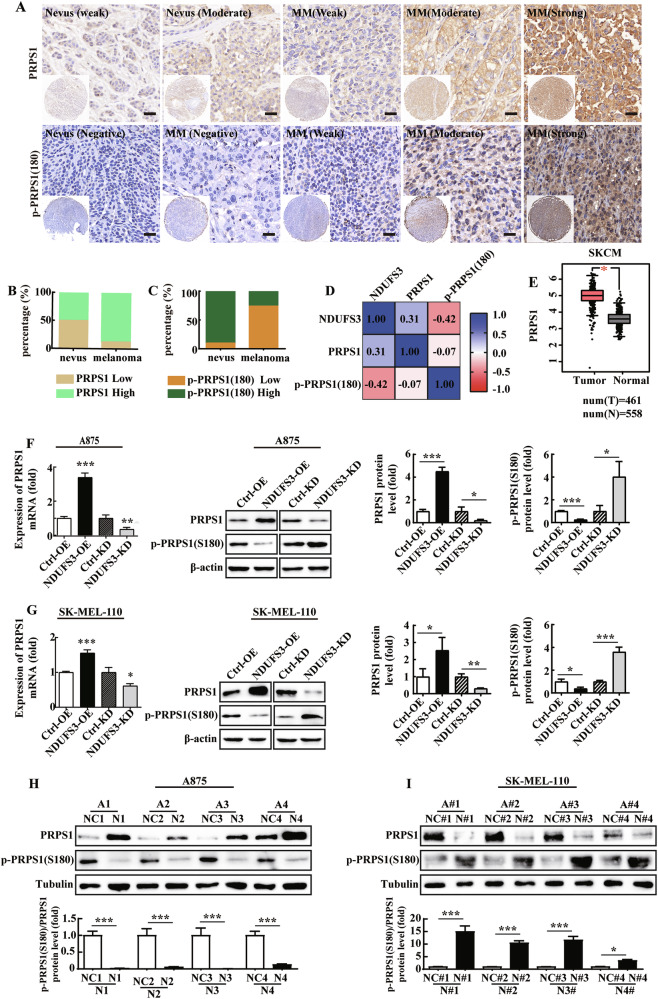
NDUFS3 promotes melanoma cell growth by increasing the activity of PRPS1. **A** IHC analysis of PRPS1 (top panel) and p-PRPS1 (180) (bottom panel) expression in normal nevi and melanoma tissues. Images of PRPS1 and *p*-PRPS1 (180) expression levels are shown (400 ×). Scale bar=50 µm. **B** Percentage of normal nevus and melanoma tissues with low and high expression of PRPS1. **C** Percentage of normal nevus and melanoma tissues with low p-PRPS1(180) expression and high p-PRPS1(180) expression. **D** Pearson correlation analysis was used to analyze the expression of NDUFS3, PRPS1 and p-PRPS1(180) in melanoma samples. **E** The mRNA level of PRPS1 in SKMs and adjacent tissues based on the GEPIA database. **F** PRPS1 mRNA expression in NDUFS3-overexpressing or NDUFS3-knockdown A875 and SK-MEL-100 cells. **G** Protein levels of PRPS1 and p-PRPS1(180) in NDUFS3-overexpressing or NDUFS3-knockdown A875 and SK-MEL-100 cells. **H, I** The protein level of PRPS1 and p-PRPS1 (S180) in tumor tissues of NDUFS3 transplanted tumors. Each bar represents 3 independent experiments, each carried out in triplicate, mean ± SD. *p* values were calculated using Student’s *t* test. *p < 0.05, **p < 0.01, ***p < 0.001; ns indicates no significant difference.

First, we evaluated the correlation between NDUFS3, PRPS1, and p-PRPS1(S180) expression levels of the same melanoma tissue microarray (MME1004i) and pathological parameters. Pictures of the IHC staining are shown in Fig. 7A. The results of IHC analysis of PRPS1 and p-PRPS1(S180) expression in melanoma are summarized in Table 1. Furthermore, the expression levels of PRPS1 were significantly elevated in 87.6% of 89 melanoma samples and 50% of 10 nevi samples (Fig. 7B). The expression of p-PRPS1(S180) was significantly downregulated in 74.2% (66/89) of the melanoma specimens, whereas 90.0% (9/10) of the nevi exhibited increased expression of p-PRPS1(S180) (Fig. 7C). In addition, Pearson correlation analysis indicated that the protein expression level of NDUFS3 was positively correlated with that of PRPS1 (Pearson correlation coefficient r = 0.31, *P* < 0.001) and negatively correlated with that of p-PRPS1(S180) in melanoma samples (Pearson correlation coefficient r = −0.42, *p* < 0.001) (Fig. 7D). Subsequently, the results of analyzing the GEPIA database indicates that the mRNA level of PRPS1 is upregulated in melanoma samples (Fig. 7E).

Subsequently, compared with the control cells, overexpression of NDUFS3 markedly enhanced the expression levels of both PRPS1 mRNA and protein, while concurrently reducing the expression of the p-PRPS1(S180). Conversely, upon knocking down NDUFS3, there was a notable decrease in the expression levels of PRPS1 mRNA and protein, coupled with an increase in the expression of p-PRPS1(S180) (Fig. 7F, G). Meanwhile, in vivo findings demonstrated that, relative to the respective control group, tumor tissues derived from melanoma cells overexpressing NDUFS3 exhibited elevated PRPS1 expression and reduced p-PRPS1(S180) expression. Knocking down NDUFS3 in these cells led to a decrease in PRPS1 expression and an increase in p-PRPS1(S180) expression (Fig. 7H, I). This result is consistent with the clinical samples and in vitro experimental analysis results.

Taken together, these data suggested that NDUFS3-mediated melanoma progression was associated with changes in the activity of the nucleotide metabolic enzyme PRPS1.

### AMPK phosphorylates PRPS1(S180) to inhibit melanoma cell proliferation

**Fig. 8:**
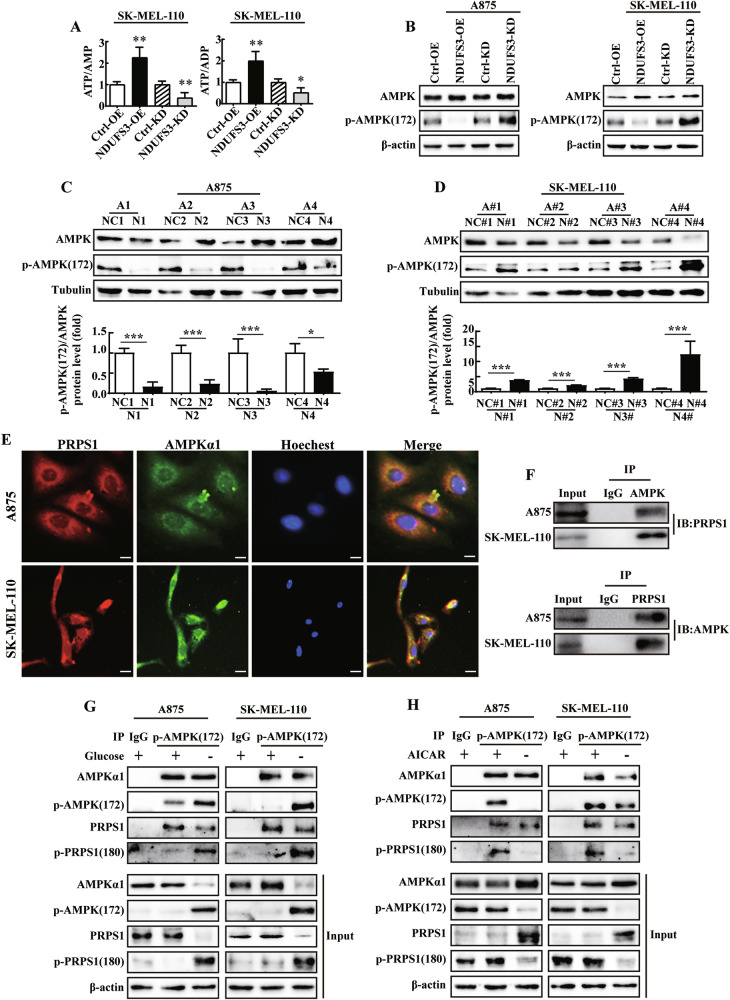
PRPS1 is the phosphorylation substrate of AMPK. **A** ATP/AMP and ATP/ADP ratios in SK-MEL-110-overexpressing and control SK-MEL-110-overexpressing and NDUFS3-knockdown SK-MEL-110 cells. These data represent three independent experiments, each carried out in triplicate. Each bar represents the mean ± SD. *p* values were calculated by using Student’s t test. *p < 0.05; **p < 0.01. **B** AMPK and p-AMPK (172) levels were measured in NDUFS3-knockdown or NDUFS3-overexpressing A875 and SKMEL-110 cells. The protein levels of AMPK and p-AMPKα1(T172) in the subcutaneous tumor tissues of A875 NDUFS3-overexpressing nude mice and control group mice (**C**) and SK-MEL-110 NDUFS3-KD nude mice and control group mice (**D**). The image analysis software Image J was used to analyze the grayscale values of Tubulin and AMPK and p-AMPKα1(T172) in various samples. Then, we calculated the ratio of AMPK or p-AMPKα1(T172) to Tubulin and normalized the ratios of the control group to assess the changes in NDUFS3 expression in both the control and experimental groups of mice. **E** AMPK and PRPS1 colocalization in A875 (top panel) and SK-MEL-110 (bottom panel) cells was detected by immunofluorescence. PRPS1 (red), AMPK (green), and DAPI (blue) (magnification, 100×). **F** Co-IP analysis of A875-WT and SK-MEL-110-WT cells was performed using anti-AMPK, PRPS1 or IgG antibodies. Quantitative Co-IP analysis of WT melanoma cells treated with complete medium or glucose deprivation medium for 6 h (**G**) or treated with or without AIACR for 6 h (**H**) was performed.

AMPK is the most important energy regulatory factor that can sense the balance of ATP/AMP and ATP/ADP in the cell [40]. Following the overexpression of NDUFS3, the ATP/AMP and ATP/ADP increased (Fig. 8A). However, ATP/AMP and ATP/ADP decreased in NDUFS3 knockdown cells (Fig. 8A). Interestingly, increased p-AMPK(T172) was observed in NDUFS3-knockdown cells, whereas p-AMPK(T172) was decreased in NDUFS3-overexpressing melanoma cells (Fig. 8B). Compared with those in control cells, the p-AMPK(T172)/AMPK ratio in tumor tissues overexpressing NDUFS3 was reduced, while the p-AMPK(T172)/AMPK ratio in tumor tissues with NDUFS3 knockdown was increased (Fig. 8C, D). Therefore, we speculated that NDUFS3 may affect the proliferation of melanoma cells through AMPK phosphorylated PRPS1 mediated nucleotide metabolism.

To test this hypothesis, IF and Co-IP analyses were initially performed to explore whether AMPK can bind directly to PRPS1. Representative images indicated that AMPKα1 could colocalize with PRPS1 (Fig. 8E). The Pearson correlation coefficient (PCC) and Mander’s overlap coefficient (MOC) were used to evaluate the colocalization factors [41]. The IF results suggested that PRPS1 was positively correlated with AMPKα1 (A875:PCC = 0.804, MOC = 0.81; SK-MEL-110:PCC = 0.940, MOC = 0.950). Endogenous AMPKα1 was immunoprecipitated from A875 or SK-MEL-110 cell lysates using a PRPS1 antibody (Fig. 8F). To further elucidate the potential of PRPS1 to act as a substrate of AMPKα1, the expression levels of AMPKα1, p-AMPKα1(T172), PRPS1 and p-PRPS1(S180) were determined in melanoma cells that were glucose deprived or treated with AIACR, an AMPK activator that can markedly increase the level of phosphorylated AMPK. Previous studies have shown that p-PRPS1(S180) is the only site for AMPK phosphorylation of PRPS1 [42]. A prominent increase in p-AMPK(T172) was observed in the melanoma cell lines following glucose deprivation (Fig. 8G) or incubation with AIACR (Fig. 8H) for 6 h. In addition, the expression levels of p-PRPS1(S180) were also increased (Fig. 8G, H). These results suggested that AMPK could directly interact with PRPS1.

### NDUFS3 promotes melanoma cell proliferation by regulating PRPS1 enzyme activity through AMPK

**Fig. 9:**
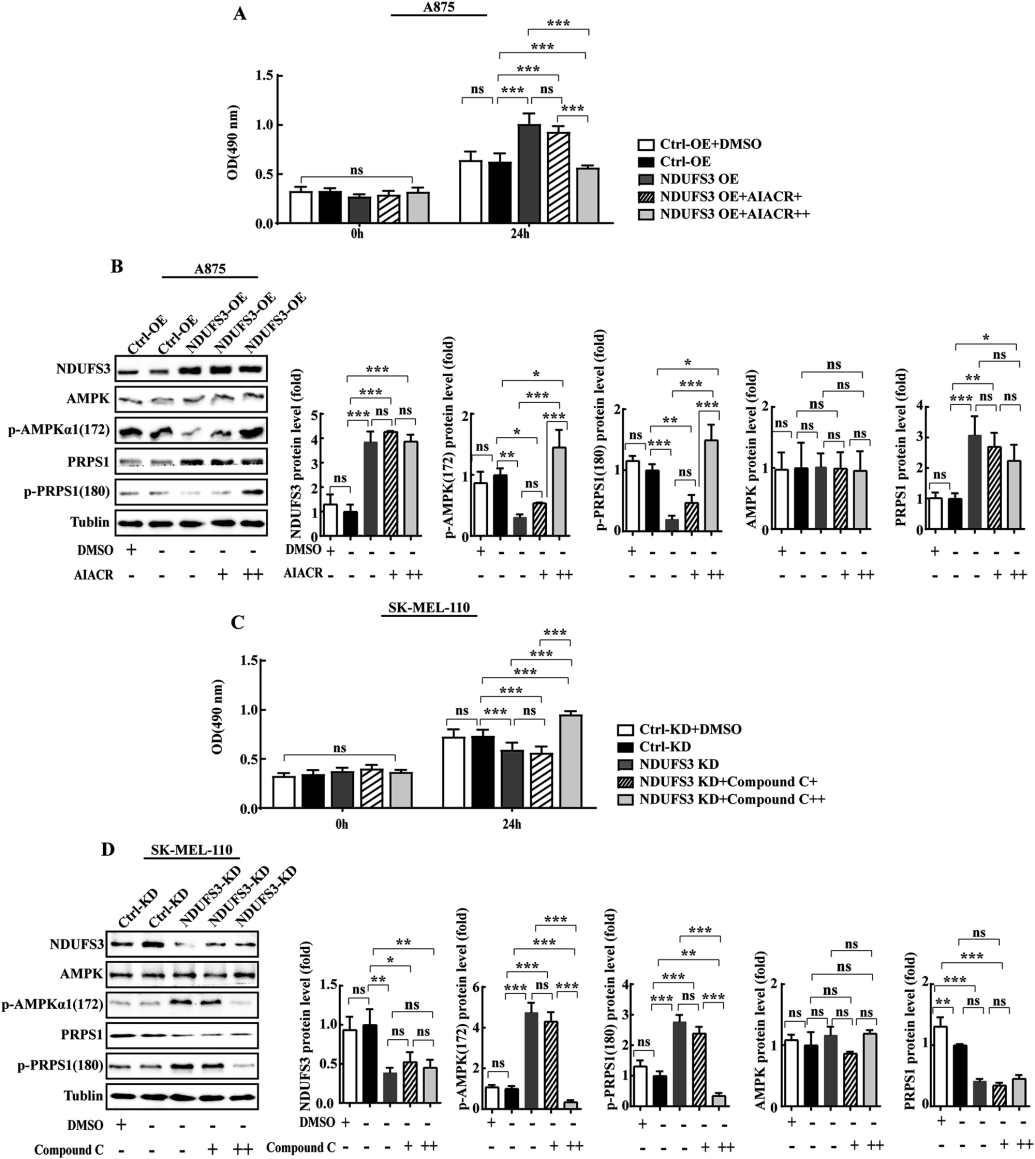
NDUFS3 inhibits melanoma proliferation by downregulating PRPS1 activity through AMPK. After incubation of NDUFS3-overexpressing A875 and control cells with AIACR (5 mM, 10 mM), the cell proliferation rate (**A**) was measured by MTS, and the protein levels of AMPK, p-AMPKα1 (T172), PRPS1 and p-PRPS1 (S180) were detected by western blotting (**B**). DMSO, 10 mM DMSO; AIACR + , 5 mM AIACR; AIACR + + , 10 mM AIACR. **C, D** After incubation of NDUFS3 knockdown SK-MEL-110 cells and control cells with compound C (10 µM, 20 µM), the cell proliferation rate (**A**) was measured by MTS, and the protein levels of AMPK, p-AMPKα1 (T172), PRPS1 and p-PRPS1 (S180) were detected by western blotting (**B**). DMSO + , 20 µM DMSO; Compound C + , 10 µM Compound C; Compound C + + , 20 µM Compound C. The data from three independent experiments are expressed as the mean ± SD, each carried out in triplicate. and were analyzed with two-way ANOVA. *p < 0.05, **p < 0.01, ***p < 0.001, *ns* indicates no statistical significance.

The effects of the association of AMPK-PRPS1 with NDUFS3-mediated melanoma cell proliferation were examined. We incubated NDUFS3-OE A875 cells with AIACR for proliferation phenotype recovery experiments. First, after treating A875 NDUFS3-OE and Ctrl-OE cells with the AMPK activator AIACR (5 mM, 10 mM) or DMSO for 48 h, the MTS results showed that the cell proliferation rates, from highest to lowest, were NDUFS3-OE + AIACR 5 mM>Ctrl-OE≈Ctrl-OE + DMSO > NDUFS3- OE + AIACR 10 mM (Fig. 9A). The AMPK activator AIACR (10 mM) inhibited the ability of NDUFS3 to promote the proliferation of melanoma cells. Moreover, AIACR stimulation increased the expression of p-AMPK(T172) and p-PRPS1(S180) in NDUFS3-OE cells, and the higher the concentration of AIACR was, the greater the expression levels of p-AMPK(T172) and p-PRPS1(S180), and the lower the proliferation rate of NDUFS3-OE A875 cells. Moreover, the expression levels of AMPK and PRPS1 in NDUFS3-OE A875 cells were greater than those in Ctrl-OE cells, but AIACR treatment did not alter the expression of AMPK or PRPS1 in NDUFS3-OE A875 cells (Fig. 9B).

In addition, we incubated NDUFS3-KD SK-MEL-110 cells with an AMPK inhibitor (Compound C) for proliferation phenotype recovery experiments. First, after SK-MEL-110 NDUFS3-KD and Ctrl-KD cells were treated with the AMPK inhibitor Compound C (10 µM, 20 µM) or DMSO for 48 h, the proliferation ability of the cells was detected using MTS. The results showed that the cell proliferation rates, from highest to lowest, were as follows: NDUFS3-KD+Compound C 20 µM>Ctrl-KD + DMSO≈Ctrl-KD > NDUFS3-KD+Compound C 10 µM (Fig. 9C). Compound C (20 µM) reversed the inhibitory effect of NDUFS3 knockdown on melanoma cell proliferation. In addition, western blot results showed that Compound C can inhibit the levels of p-AMPK(T172) and p-PRPS1(S180) in NDUFS3-KD cells, and the higher the concentration of Compound C was, the lower the expression levels of p-AMPK(T172) and p-PRPS1(S180), and the greater the proliferation rate of NDUFS3-KD cells. Moreover, the expression levels of AMPK and PRPS1 in NDUFS3-KD cells were lower than those in Ctrl-KD cells, but Compound C treatment did not alter the expression of AMPK or PRPS1 in NDUFS3-KD cells (Fig. 9D).

Collectively, these results suggested that NDUFS3 downregulates/upregulates melanoma cell proliferation by promoting/inhibiting AMPK phosphorylation and reducing/promoting PRPS1 activity.

## Discussion

Accumulating evidence has indicated different preferences of cancer cells for aerobic glycolysis (typical Warburg effect) or OXPHOS (nontypical Warburg effect) [32, 43]. Aerobic glycolysis in tumors is characterized by increased glucose uptake and lactate production [1, 2, 44]. Tumors in a high-OXPHOS state exhibit increased expression of genes related to respiratory chain components, enhanced mitochondrial respiration and increased antioxidant defense [32, 43, 45]. These outcomes are associated with decreased lactate production [32, 43, 45]. In the present study, high OXPHOS metabolic heterogeneity, which was based on the overexpression of NDUFS3, was detected in melanoma cells. These effects were associated with AMPK activity and purine nucleotide metabolism.

NDUFS3 is a core subunit that is highly conserved during evolution and is considered necessary for CI assembly [21, 46]. The expression levels of NDUFS3 are heterogeneous in different tumor types. Low expression levels of NDUFS3 were detected in kidney cancer [47], serous ovarian cancer [48] and human mammary carcinoma (HMC-1) [49]. The expression levels of NDUFS3 may be associated with poor patient prognosis. High expression levels of NDUFS3 were noted in colorectal cancer [50], gastric cancer [51] and highly invasive breast carcinoma [25]. Our data indicated that NDUFS3 was highly expressed in melanoma cells, while its overexpression promoted highly malignant behavior and poor prognosis in melanoma patients. The upregulated or downregulated expression of NDUFS3 promoted or inhibited the proliferation of melanoma cells and melanoma xenotransplantation, respectively.

Studies have shown that NDUFS3 deficiency is associated with mitochondrial dysfunction in breast cancer [49, 52] and chromophobe renal cell carcinomas [22]. The present study confirmed that NDUFS3 deletion induced mitochondrial fragmentation and destruction in melanoma cells. Moreover, we found that increased NDUFS3 abundance increased the complexity of the mitochondrial network and promoted OXPHOS in melanoma cells. Previous studies have identified several molecular drivers of melanoma that affect aerobic glycolysis and OXPHOS, as well as the expression levels of peroxisome proliferator-activated receptor-γ coactivator-1α (PGC-1α), BRAF, and NRAS. Mutation of BRAF and NRAS and overexpression of PGC-1α in melanoma cells indicate high OXPHOS and a greater dependency of these cells on glycolysis [53, 54]. In the present study, the abundance of NDUFS3 in melanoma increased the TCA cycle and OXPHOS, while it concomitantly decreased glycolysis. In contrast to these findings, the deletion of NDUFS3 in melanoma cells downregulated OXPHOS and upregulated glycolysis, which is consistent with the findings of a recent report indicating that glycolysis was upregulated in KO NDUFS3 neurons [55]. The results of the present study further demonstrated that NDUFS3 could be used as a marker molecule of high OXPHOS activity in melanoma. Moreover, we have discovered for the first time that NDUFS3 acts as a ‘switch’ for the regulation of OXPHOS and glycolysis in melanoma.

An increase in OXPHOS, which is mediated by NDUFS3 in melanoma, results in an increase in the PPP, purine nucleotide metabolism and nucleic acid synthesis to sustain the proliferation of cancer cells (Fig. 3, Fig. 6). It is worth noting that our study has further confirmed that the abnormal expression of NDUFS3 primarily impacts the fluctuation of purine nucleotides in melanoma cells, as opposed to pyrimidine nucleotides (Fig. 6D, Fig. S4). PRPS1 is the first enzyme involved in nucleotide metabolism. It has been reported that knockdown of PRPS1 expression suppresses proliferation and promotes apoptosis in neuroblastoma [56] and human breast cancer cells [57]. We also found that in melanoma cells overexpressing NDUFS3, the levels of AMP, GMP, ADP, GDP, ATP, and GTP significantly increased (Fig. 6D). This not only demonstrates the direct synthetic effect caused by the upregulation of PRPS1 activity but also aligns with the “engine” role of PRPS1 in the purine synthesis process, which drives a large influx of precursors into the synthetic pathway to efficiently produce purine nucleotides. Furthermore, to maintain metabolic balance, the body adaptively upregulates enzymes related to purine nucleotide synthesis, such as ADSL, GMPS, IMPD1 and IMPD2 (Fig. 6E) to meet metabolic demands. The results revealed that the expression levels of NDUFS3 and p-PRPS1(S180) exhibited a direct negative correlation. Moreover, the AMPK-mediated phosphorylation of PRPPS1(S180) decreased PRPS1 activity, accompanied by the switching of PRPS1 hexamers to monomers [42].

As the major regulator of OXPHOS and mitochondrial biogenesis [58], AMPK is also the most important energy regulator and can sense the balance of the AMP/ATP and ADP/ATP ratios in cells [40]. Under low-energy conditions, AMPK phosphorylates various enzymes to increase ATP generation and decrease ATP consumption [59, 60]. In the present study, we found that NDUFS3 mediated metabolic changes in glucose metabolism and OXPHOS, and cells produced more ATP, thereby inhibiting AMPK activity (Figs. 7A, 6B). These data suggested that AMPK is an effective downstream effector of NDUFS3. In addition, we identified that high OXPHOS activity is dependent on the NDUFS3-AMPK axis in melanoma.

Furthermore, the application of an AMPK activator (AIACR) and inhibitor (compound C) was used to examine the correlation between NDUFS3 expression and AMPK and PRPS1 activity. Following treatment with the AMPK inhibitor, NDUFS3-knockdown melanoma cells exhibited increased cell proliferation, possibly due to the downregulation of p-AMPK(T172) expression and the increase in PRPS1 activity. Melanoma cells overexpressing NDUFS3 were incubated with an AMPK activator, leading to cell growth inhibition, which may be due to increased p-AMPK(T172) expression and decreased PRPS1 activity. Therefore, the present study indicated that NDUFS3 could coregulate the activity of p-PRPS1(S180) and promote the growth of melanoma tumors by inhibiting the levels of p-AMPK (T172).

## Materials and methods

### Immunohistochemistry and immunohistochemical analysis

The tissue microarrays were purchased from xi,anTaibosi Biological Technology Co., Ltd. The antibodies used were the following: anti-NDUFS3(Abcam, #ab110246), anti-PRPS1(Proteintech, #15549-1-AP), customized anti-p-PRPS1(180)(Wuhan Maisi Biotechnology Co., Ltd.) and Ki67(Bioss, #bs-2130R). The specimens were stained with Envision Detection Kit/DAB(DAKO A/S, #GK500705) and counterstained with hematoxylin(ZLI 9615, ZSGB-BIO). The images were obtained using the panoramic slice scanner.

Immunohistological assessment was performed by detecting the expression signal of NDUFS3, PRPS1, p-PRPS1(180) and Ki67 using image reader Caseviewer 2.4 (400× magnification). The immunohistochemical score was carried as follows: The staining intensity was marked as 0 points (negative intensity), 1 point (weak intensity), 2 points (moderate intensity) and 3 points (strong intensity). The percentage of positive stained cells in each region was graded as follows: 0 points (≤5%), 1 points (6–25%), 2 points (26–50%), 3 points (51–75%) and 4 points (≥76%). Subsequently, the staining intensity was multiplied by the percentage of stained cells, which was equal to the expression fraction of the detection factors in melanoma. Finally, the samples with a final score of 0 points was recorded as negative (-), whereas a score of 1–4 points as weak (+), a score of 5–8 marked as moderate (++), and a score of 9–12 points as strong (+++). The final scores below 5 points were classified as low expression, while the final scores higher than 4 points were recorded as high expression. All results were evaluated by two pathologists with the double-blind method.

### Bioinformatics analysis

The acquisition of the NDUFS3 mRNA expression data from 458 patients with pancreatic cancer was performed using the TCGA database. The survival curves of melanoma patients with NDUFS3 high or NDUFS3 low were compared by the log-rank test. GEPIA was used to explore the expression levels of PRPS1 in 461 melanoma and 558 normal samples.

### Cell culture and establishment of stable cell lines

The human melanoma cell lines A875 and SK-MEL-110 were obtained from the Cell Resource Center, Institute of Basic Medical Sciences, Chinese Academy of Medical Sciences [61]. All cells were cultured in DMEM (Gibco, #11965092) supplemented with 10% fetal bovine serum(BI, #04-001ACS) at 37 °C in the presence of 5% CO_2_.

The stable transfectants of NDUFS3 overexpression and knockdown A875 and SK-MEL-110 cell lines were established using lentiviral expression vectors, which were obtained from Ji Kai gene Chemical Technology Co., Ltd. A875 and SK-MEL-110 cells in the logarithmic growth phase were seeded into 6-well plates. A total of 6 × 10^4^ cells were incubated per well and cultured overnight. According to MOI and cell counts, an appropriate amount of infection enhancement solution and virus were added. The cells were cultured for 24 h and replaced with fresh complete medium. On the third day, the cells were treated with different concentrations of puromycin. The rate of GFP-positive cells observed under a fluorescence microscope was adjusted to be higher than 95% and qPCR and western blotting methods were used to identify and obtain stable transfectant cell lines.

### RNA isolation and RT-PCR analysis

Total RNA was collected using TRIzol reagent(Takara, #9109). Complementary DNA was performed using the thermo RT Kit. QPCR was synthesized using SYBR-Green Master(ROX)(Roche, #04913914001) on a real-time PCR, according to the manufacturer’s instructions. The primer sequences as Table S1.

### Western blot analysis

The cells were lysed in RIPA buffer(Solarbio, cat#R0010), containing protease and phosphatase (1 v:100 v) inhibitors on ice for 30 min. The protein lysates were centrifuged at 12,000 g, at 4 °C for 10 min. The protein contents were evaluated using a BCA^TM^ Protein Assay Kit(Applygen, #P1511). The proteins were separated using 10% SDS-polyacrylamide gel electrophoresis and transferred to polyvinylidene fluoride (PVDF) membranes(Millipore, #IPVH00010). The membranes were closed with 5% BSA for 2 h at ambient temperature. The primary antibodies were incubated with the corresponding membranes at 4 °C overnight. The membranes were incubated for 1 h at room temperature. Subsequently, the membranes were washed four times (5 min each time), with 1×TBST on the shaker and incubated with secondary antibodies at room temperature for 1 h. Finally, the chemiluminescent signals were amplified using the chemiluminescence reagent ECL kit(Advansia, #K-12045-D50) and a high-quality image was acquired using the Bio-Rad ChemiDoc XRS system.

The primary antibodies used were the following: Mouse monoclonal anti-NDUFS3(Abcam, #ab110246), monoclonal anti-PRPS1(Proteintech, #15549-1-AP), customized anti-p-PRPS1(180)(Wuhan Maisi Biotechnology Co., Ltd.), anti-AMPKα1(CST, #2532), anti-p-AMPKα1(172)(CST, #2535), anti-β-actin(Bioss, #0061 R), Mfn2(Zenbio, #340604), DRP1(Zenbio, #340336), P-AMPK(CST, #2535) and NDUFS1(Zenbio, #R27070). The secondary antibodies used were the following: Goat anti-mouse IgG-HRP(SAB, #L3032-2) and anti-rabbit IgG-HRP(Abcam, #ab6721).

### Coimmunoprecipitation assay

The cells were lysed in immunoprecipitation lysis buffer (Beyotime, P0013D) on ice for 30 min, followed by centrifugation at 12,000 g, at 4 °C for 10 min to remove the precipitate. The protein lysate was divided into four parts, of which one was immunoprecipitated with anti-AMPKα1(Santa, #sc-398861) antibody and the other with anti-PRPS1(Proteintech, #15549-1-AP) antibody. The third part was incubated with IgG at 4 °C overnight with constant rotation for 8 h and the remaining sample was stored at −20 °C. The fourth part is stored at −20 °C as input for future use. Following rinsing with PBS, Protein G Sepharose beads(Roche, #11243233001) were added to the protein lysates, which were immunoprecipitated at 4 °C overnight with constant rotation. Following washing with PBS, the protein lysates were collected and heated at 100 °C for 10 min, followed by immunoblotting.

### Cell proliferation assays

Cell proliferation was detected by the EDU assay Kit(Beyotime, #C0078S) according to the manufacturer’s instructions. Appropriate amounts of the cells were seeded into 24-well plates and cultured in liquid medium overnight. Following 24 h, the cells were incubated with 100 μL complete medium containing 10 μM EDU solution for 2 h. Subsequently, the cells were fixed with 4% paraformaldehyde for 15 min and washed three times with PBS. The cells were treated with 0.3% Triton-100 for 15 min, washed three times with PBS once, incubated with 200 μL click reaction solution for 30 min and washed three times with PBS for a final time. A total of 200 μL Hoechst solution (Hoechst: PBS = 1:5000) was added to the cells for 15 min, which were washed three times by PBS. Finally, the cells were observed by a Leica DMI6000B microscope to acquire the images.

The MTS cell proliferation kit(Promega, #CTB169) was used to examine cell proliferation according to the manufacturer’s instructions. The cells were seeded into 96-well plates and cultured overnight. According to the experimental design, the cells were treated with AMPK inhibitor compound C (MedChemExpress, #866405-64-3) or AMPK activator AIACR(MedChemExpress, #2627-69-2) or DMSO(Sigma, #0500095) for 24 h and incubated with 10 μL MTS at 37 °C for 2 h. The absorbance was measured using a microplate reader.

### Mitochondrial morphological assay

The cells were seeded into a confocal culture dish(ThermoFisher, #150682), cultured overnight and finally treated with 100 μL (800 nM) MitoTracker® Deep Red(CST, #8778) for 30 min at 37 °C. Following incubation, the cells were fixed with 4% paraformaldehyde for 15 min, and subsequently rinsed with PBS 3 times. High-quality images were obtained with a Leica TCSSP5 confocal microscope.

### Mitochondrial membrane potential analysis

The exponential growth stage cells were prepared into suspension cells and cultured overnight in 6-well plates. The cells were subsequently incubated using 1% TMRM(Invitrogen, #I34361) in fresh medium (10% FBS) for 30 min at 37 °C. Following washing once with PBS, the cells were digested by trypsin, and collected to detect the fluorescence intensity by flow cytometry.

### OCR and ECAR measurements

The xtracellular acidification rate (ECAR)(Bestbio, #BB48311) were measured by a fluorescence microplate reader. A total of 0.8 × 10^5^ cells/well were incubated in black 96-well plates in the presence of 100 μL complete medium overnight. Prior to detection, the cells were incubated in a non-CO_2_ incubator for at least 6 h. Following washing with buffer, 90 μL fresh medium was added to the black 96-wells and subsequently 10 μL BBcellProbe® P16 solution was added. Finally, the continuous dynamic reaction was achieved for 1 h. OCR analysis was performed using 1 × 10^4^ cells/well, which were seeded into 96-well plates and incubated overnight. The cells were washed with PBS and 10 μL BBcellprobe® R01 was mixed with 150 μL fresh medium and added to the cells. The continuous dynamic reaction was detected for 2 h.

### Lactate concentration, glucose uptake and enzyme activity analysis

The cell lines were incubated into 6-well plates at a density of 1 × 10^5^ cells per well, and cultured overnight. The cells were treated with serum-free medium for 48 h. Subsequently, complete medium was used to replace the culture medium for an additional 48 h. The cell supernatant was collected to detect lactate concentration(APPLYGEN, #E1011) and glucose uptake(Jiangsu Fiat Biotechnology Co., Ltd., #MM-12907H1) according to the experimental protocol used.

Concomitantly, the cells were collected to measure the activity of PDH(Sigma, #MAK183), PK(Solarbio, #BC0540), HK(Solarbio, #BC0740) and G6PD(Genmed, GMS70013.1) according to the manufacturer’s protocol.

### Metabolite quantification and metabolomics analysis

NDUFS3 overexpression or knockdown SK-MEL-110 cells were seeded in a 75 cm^2^ tube bottle. The cells were harvested (1 × 10^7^ cells per tube, 7 tubes per sample) for metabolomic analysis. Quantitative analysis was performed by assessing the samples with LC-MS/MS at Shanghai Applied Protein TechnologyCo. Ltd.

### Immunofluorescence staining

The images of immunofluorescence staining were acquired with a Leica TCSSP5 confocal microscope (×400 magnification) and analyzed by image J. The cells were immunostained with the following antibodies: Anti-AMPKα1(Abcam, #ab110036), anti-PRPS1(Proteintech, #15549-1-AP), CoraLite488-conjugated Affinipure Goat anti-Mouse IgG(H + L)(Proteintech, #SA00013-1) and Cy3–conjugated Rabbit Anti-Goat IgG(H + L)(Proteintech, #SA00009-4).

### UDP、UTP、CTP assays

Cell lysates were prepared using RIPA lysis buffer to extract total cellular protein, and protein concentration was determined using the BCA method. Following the manufacturer’s guidelines, ELISA kits were used to measure the levels of UDP (HuaBang, HB-P36021R), UTP (HuaBang, HB-P95544R), and CTP (HuaBang, HB-P35874R).

### Soft agar colony formation assay

5000 cells per well were seeded into a 6-well plate containing 0.5% bottom agar and 0.35% top agar, and cultured for 14 days. The size and number of cell colonies were observed under an inverted microscope (using a 100× objective lens), and images were captured.

### CI Enzyme activity and CI content assay

Extract cell mitochondria following the instructions provided by the reagent supplier (Abcam, #ab110170). Then, detect the activity of CI according to the instructions of the reagent supplier (Abcam, #ab109721). Finally, measure the content of CI following the instructions from the reagent supplier (Tianjin Yisenyuan Biotechnology Co., Ltd, YSH108819).

### Flow cytometry

To conduct cell cycle assays, cells are initially inoculated into a 6-well plate, followed by digestion and centrifugation. Subsequently, the cells are slowly added dropwise to 75% pre-cooled ethanol and fixed at 4 °C for 24 h. The following day, the cells are collected via centrifugation, stained using a cycle kit (Biotech, #FXP0211), and analyzed by flow cytometry (BD FACSCelestaTM flow cytometry, BD) with data processed using FlowJo software.

### Animal models

Six-week-old, male, BALB/c nude mice were obtained from the Laboratory Animal Center of Kunming Medical University and randomly divided into two groups, with six mice in each group, for the establishment of subcutaneous xenograft tumor models. A 100 µL PBS solution containing 5 × 10^6^ cells (divided into NC-OE and OE groups, as well as NC-KD and KD groups) was subcutaneously injected into the same side of each mouse. Tumor volumes were observed and recorded daily. When the subcutaneous tumors met the experimental requirements and the maximum tumor diameter did not exceed 1.5 cm, the mice were euthanized. The tumor volume was calculated using the formula: Volume = 1/2 × Length × Width².

### Statistical analysis

All statistical analyses were performed using SPSS 18.0 software and the graphs were carried out using GraphPad Prism® version 8.0. The data are presented as mean ± SD. The differences between the groups were detected by the Student t-test, one-way ANOVA or the χ^2^ test as described earlier. A value of *P* < 0.05 was considered to indicate statistically significant differences.

## Supplementary information

Supplementary Figure S1

Supplementary Figure S2

Supplementary Figure S3

Supplementary Figure S4

Supplementary Figure S5

Supplementary Table S1

Supplementary figure and table legends

Original Data Files

## Data Availability

This study does not involve datasets requiring public deposition. All experimental procedures and results are fully described in the manuscript.
