# Supplementary figures and images for: NDUFS3 promotes proliferation via glucose metabolism reprogramming inducing AMPK phosphorylating PRPS1 to increase the purine nucleotide synthesis in melanoma
Source: Cell Death Differ. 2025 May 22;32(12):2193–209. doi: 10.1038/s41418-025-01525-4 (PMC12669717; doi:10.1038/s41418-025-01525-4)

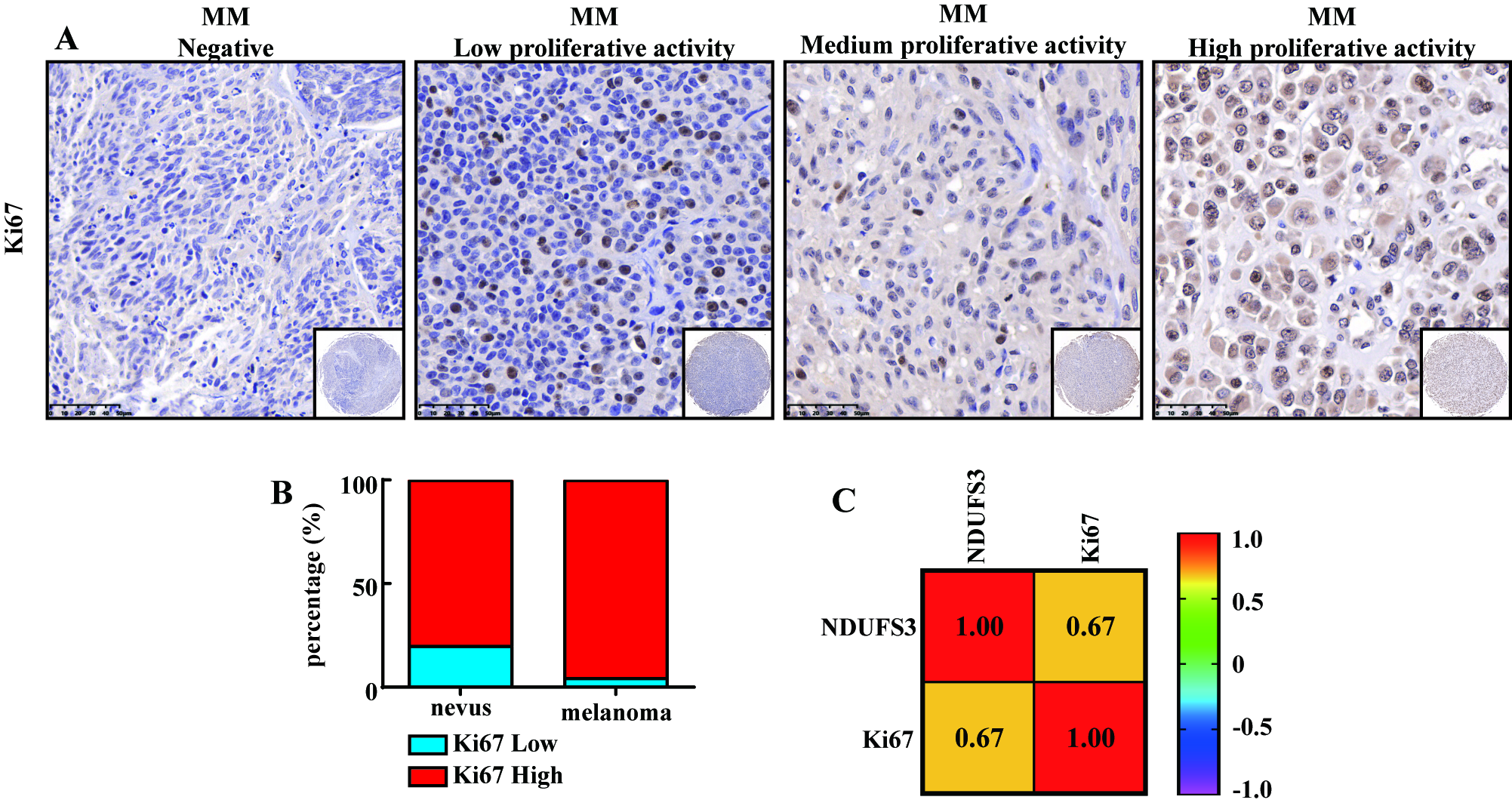

Supplement: Supplementary file 1 — Supplementary Figure S1 [file 41418_2025_1525_MOESM1_ESM.tif]

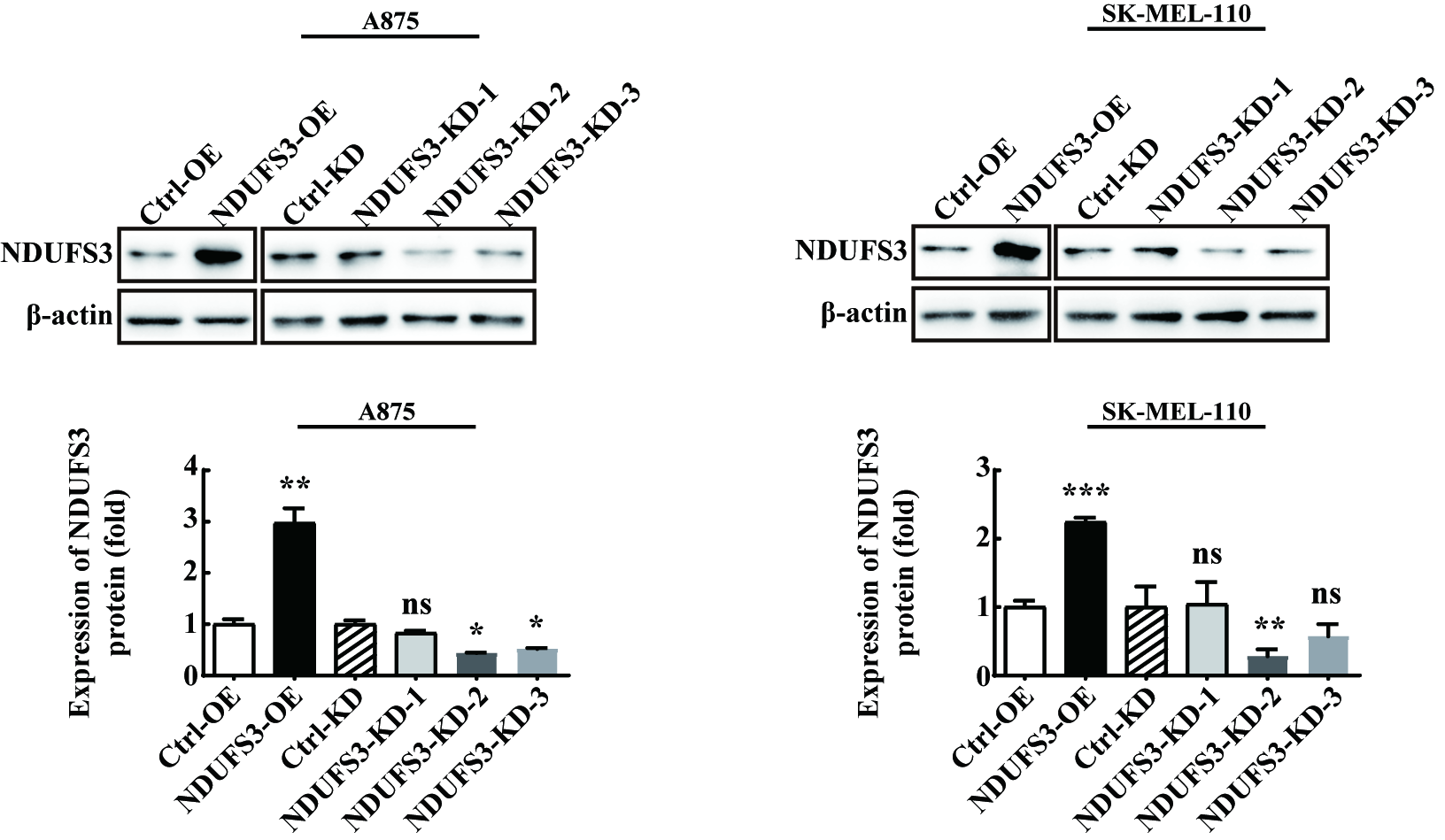

Supplement: Supplementary file 2 — Supplementary Figure S2 [file 41418_2025_1525_MOESM2_ESM.tif]

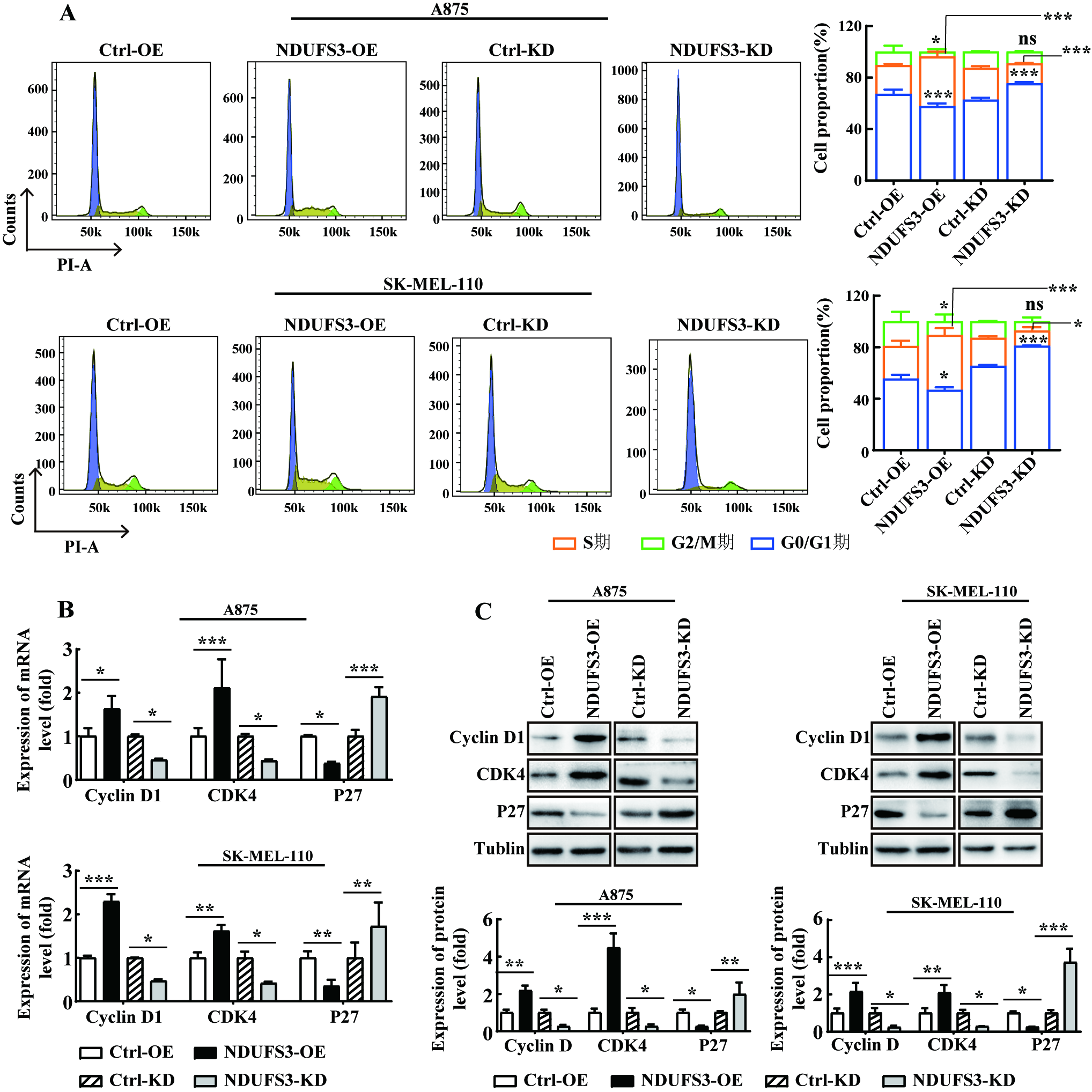

Supplement: Supplementary file 3 — Supplementary Figure S3 [file 41418_2025_1525_MOESM3_ESM.tif]

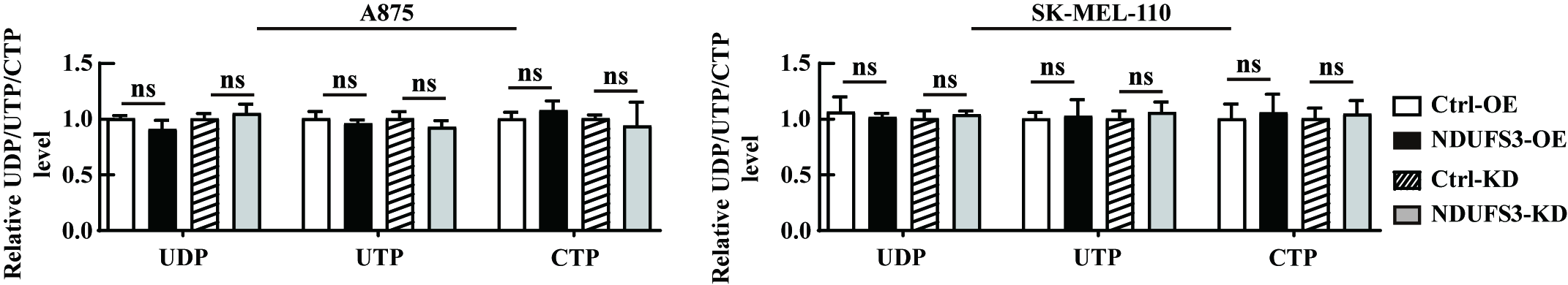

Supplement: Supplementary file 4 — Supplementary Figure S4 [file 41418_2025_1525_MOESM4_ESM.tif]

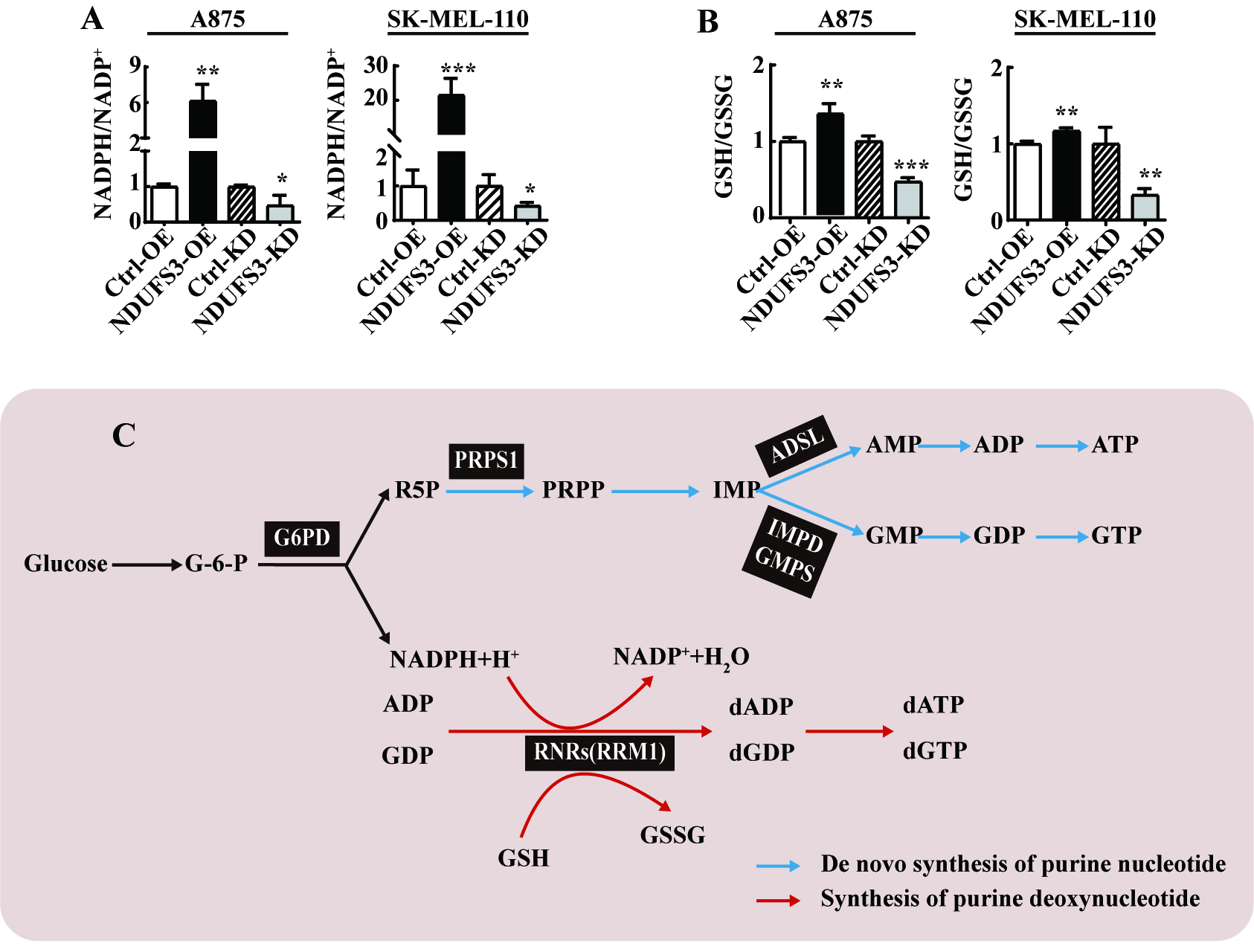

Supplement: Supplementary file 5 — Supplementary Figure S5 [file 41418_2025_1525_MOESM5_ESM.tif]
