# Supplementary material for: NDUFS3 promotes proliferation via glucose metabolism reprogramming inducing AMPK phosphorylating PRPS1 to increase the purine nucleotide synthesis in melanoma
Source: Cell Death Differ. 2025 May 22;32(12):2193–209. doi: 10.1038/s41418-025-01525-4 (PMC12669717; doi:10.1038/s41418-025-01525-4)
Supplement: Supplementary file 6 — Supplementary Table S1 [file 41418_2025_1525_MOESM6_ESM.pdf]

Table S1 The sequence of the primers for Lentivirus Expression Vector and qPCR

Gene	primer
LV-NDUFS3-RNAi-1	ACCCTGATCTAAGAAGGAT
LV-NDUFS3-RNAi-2	TGTGAAGACCTACACAGAT
LV-NDUFS3-RNAi-3	CAAGTATGTCCAACAAGTT
LVCON	TTCTCCGAACGTGTCACGT
NDUFS3	F: 5'CACCCGCTTCACTTCATC-3'
	R: 5'-TCTTCTTTGCTAACCACCCT-3'
G6PD	F: 5'-TGGAGAATGAGAGGTGGGA-3'
	R: 5'-TGCTGGTGGAAGATGTCG-3'
GPI	F: 5'-CGTGGGTTTTGACAACTTCG-3'
	R: 5'-CGGGTTCCAGATTTGGTGAT-3'
RPE	F: 5'-GCAACACACGGCATATAGAA-3'
	R: 5'-AAACAAAAGCCAGACAAACC-3'
RPEL1	F: 5'-TCGGGAGAATGGGATGAAGG-3'
	R: 5'-CCAAAGATGGGAACTGGGT C-3'
TKTL1	F: 5'-TGCACGATCTTCTACCCA-3'
	R: 5'-CCTGTCCGATCTCAAAGC-3'
TKT	F: 5'-CTATGTTTCGGTCAGTCCCC-3'
	R: 5'-ACCTGGTCATCCTTGCTCTT-3'
IMPD1	F: 5'-CAGATTGAGGGTGGTGTCC-3'
	R: 5'-GAGGCTGTGCCCAAAAGT-3'
IMPD2	F: 5'-CCGGACAGACCTGAAGAA-3'
	R: 5'-CGAGCAAGTCCAGCCTAT-3'
ASS1:	F: 5'-TCGTGCATCCTCGTGTG-3'
	R: 5'-CCTTCTTCCTGGCTTCCTC-3'
GMPS	F: 5'-GAGTCAAAGCCTGCACAAC-3'
	R: 5'-GGAGATTCCACACACGTAACT-3'
ADSL	F: 5'-CTCCTGGCAAACCTCAAG-3'
	R: 5'-TAGCGGGTCCATGACAA-3'
PRPS1	F: 5'-CGTTGTTGATGCGAGAAA-3'
	R: 5'-ATGGTGCTTGTGGGAGAT-3'

AMPKα1	F: 5'-TTGAAACCTGAAAATGTCCTGCT-3'
	R: 5'-GGTGAGCCACAACTTGTTCTT-3'
RRM1	F: 5'-GCCGCCAAGAACGAGTCAT-3'
	R: 5'-AGCAGCCAAAGTATCTAGTTCCA-3'
Mfn2	F: 5'- 3': GAAGTGGAGAGGCAGGTGTC
	R: 5'- 3': GTCCTTCCTCTATGTGGCGG
DRP1	F: 5'- 3': AGACATCTAGCCCCGTCTCT
	R: 5'- 3': TCAGTTGGTGAGCCTGTTGT
U6	F: 5'-CTCGCTTCGGCAGCACA-3'
	R: 5'-AACGCTTCACGAATTTGCGT-3'
β-actin	F: 5'-CTACCTCATGAAGATCCTCACCGA-3'
	R: 5'-TTCTCCTTAATGTCACGCACGATT-3'
