# Supplementary material for: NDUFS3 promotes proliferation via glucose metabolism reprogramming inducing AMPK phosphorylating PRPS1 to increase the purine nucleotide synthesis in melanoma
Source: Cell Death Differ. 2025 May 22;32(12):2193–209. doi: 10.1038/s41418-025-01525-4 (PMC12669717; doi:10.1038/s41418-025-01525-4)
Supplement: Supplementary file 7 — Supplementary figure and table legends [file 41418_2025_1525_MOESM7_ESM.docx]

**Supplementary figure and table legends**

**Fig.S1 The correlation between NDUFS3 and Ki67 in melanoma**

(A) The expression of Ki67 in nevi and melanoma was detected. Optical microscopy with a 40× objective lens was used to analyze the IHC results.

(B) Based on the IHC staining score results, the number of Ki67-low(4/89) and high-Ki67(85/89) cases in melanoma and nevi(0/10) was counted and expressed as percentages.

(C) Pearson correlation analysis was used to analyze the expression of NDUFS3 and Ki67 in melanoma samples.

**Fig.S2 Establishment of melanoma cells with stable NDUSF3 overexpression or knockout**

Western blotting was used to detect the protein level in NDUFS3-overexpressing or NDUFS3-knockdown melanoma cells. Scale bars = 20 μm. The data are presented as the means ± SD and were analyzed by Student’s t test. **p* < 0.05; ***p* < 0.01; ****p* < 0.001; ns indicates no significant difference. These data represent three independent experiments, each carried out in triplicate.

**Fig.S3 NDUFS3 affects the distribution of melanoma cell cycle**

(A) The phase distribution of A875 and SK-MEL-110 cells cell cycle with NDUFS3 over-expression or knock-down and the corresponding control cells were detected by flow cytometry.

(B and C) The mRNA (B) and protein (C) expressions of Cyclin D1, CDK4 and P27 in A875 and SK-MEL-110 and the related control cells stably transfected with NDUFS3 were detected by qPCR and western blot. The data as indicated the mean±SD and was analyzed by Students t test. These data represent three independent experiments, each carried out in triplicate. **p*＜0.05, ***p*＜0.01, ****p*＜0.001.

**Fig.S4 Abnormal expression of NDUFS3 does not affect pyrimidine metabolism**

ELISA assay was performed to determine the level of UDP, UTP, and CTP after NDUFS3 overexpression and knockdown in A875 and SK-MEL-110 cells. The data are presented as the means ± SD and were analyzed by Student’s t test. ns indicates no significant difference. These data represent three independent experiments, each carried out in triplicate.

**Fig.S5 The effects of NDUFS3 on the synthesis of deoxynucleotides in melanoma**

(A-B) The content of NADPH/NADP^+^ (A) and GSH/GSSG (B) in NDUFS3 knockdown or overexpression A875 and SK-MEL-110 cells. The data represent three independent experiments, and indicate the mean ± SD and was analyzed by independent sample t test. **p* <0.05, ***p* <0.01, ****p<*0.001*.*

(C) Schematic illustration of the role of NDUFS3 in regulating deoxynucleotide synthesis in melanoma cells.

**Table S1 the sequence of the primers for Lentivirus Expression Vector and qPCR**
