# Supplementary material for: NDUFS3 promotes proliferation via glucose metabolism reprogramming inducing AMPK phosphorylating PRPS1 to increase the purine nucleotide synthesis in melanoma
Source: Cell Death Differ. 2025 May 22;32(12):2193–209. doi: 10.1038/s41418-025-01525-4 (PMC12669717; doi:10.1038/s41418-025-01525-4)
Supplement: Supplementary file 8 — Original Data Files [file 41418_2025_1525_MOESM8_ESM.pdf]

Original Data Files_original western blots

Order number	Cell	Protein	Figures
Fig.1E	A875	NDUFS3-OE	
		NDUFS3-KD	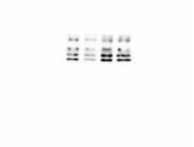
		β-actin	
	SK-MEL-110	NDUFS3	
		β-actin	
Fig.2F	A875	NDUFS3	
		Tubulin	
	SK-MEL-110	NDUFS3	
		Tubulin	
Fig.3F	A875	DRP1	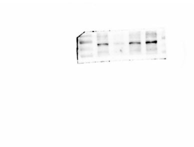

		Mfn2	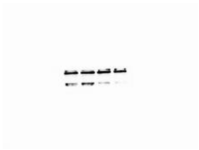
		β-actin	
	SK-MEL-110	DRP1	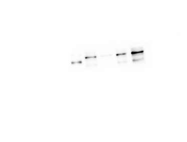
		Mfn2	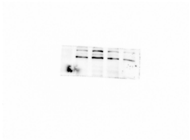
		β-actin	
Fig.6B	A875	G6PD	
		β-actin	
	SK-MEL-110	NDUFS3-OE G6PD	
		NDUFS3-KD G6PD	
		β-actin	

Fig.7F	A875	PRPS1	
		p-PRPS1(180)	
		β-actin	
Fig.7G	SK-MEL-110	NDUFS3-OE+KD-PRPS1	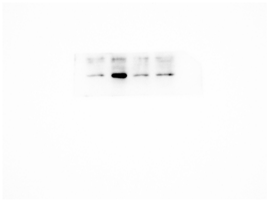
		NDUFS3-OE-p-PRPS1(180)	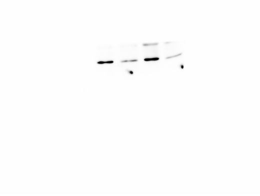
		NDUFS3-KD-p-PRPS1(180)	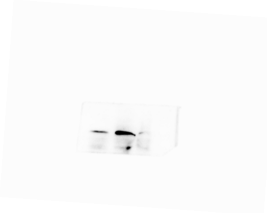
		NDUFS3-OE+KD-β-actin	
Fig.7H	A875	PRPS1	
		p-PRPS1(180)	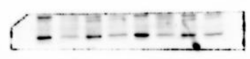

		Tubulin	
Fig.7I	SK-MEL-110	PRPS1	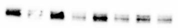
		p-PRPS1(180)	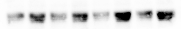
		Tubulin	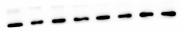
Fig.8B	A875	AMPK	
		p-AMPK(172)	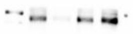
		β-actin	
	SK-MEL-110	AMPK	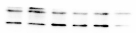
		p-AMPK(172)	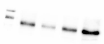

			β-actin	
Fig.8C	A875		NDUFS3-OE-AMPK	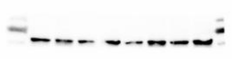
			NDUFS3-OE-p-AMPK(172)	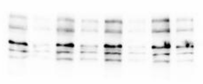
			NDUFS3-OE-Tubulin	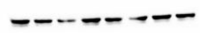
Fig.8D	SK-MEL-110		NDUFS3-OE-AMPK	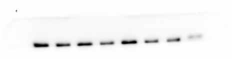
			NDUFS3-OE-p-AMPK(172)	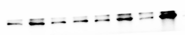
			NDUFS3-OE-Tubulin	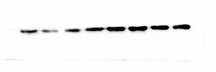
Fig.8G	A875	IP	AMPKα1	

			p-AMPK(172)	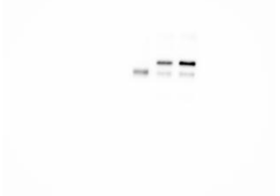
			PRPS1	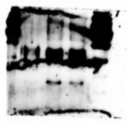
			p-PRPS1(180)	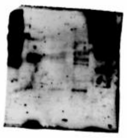
		Input	AMPKα1	
			p-AMPK(172)	
			PRPS1	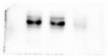
			p-PRPS1(180)	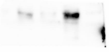
			β-actin	

<table>
<tr><td rowspan="8"></td><td rowspan="8">SK-MEL-110</td><td rowspan="4">IP</td><td>AMPKα1</td><td></td></tr>
<tr><td>p-AMPK(172)</td><td>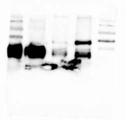</td></tr>
<tr><td>PRPS1</td><td>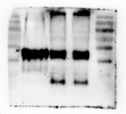</td></tr>
<tr><td>p-PRPS1(180)</td><td>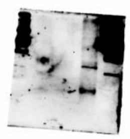</td></tr>
<tr><td rowspan="4">Input</td><td>AMPKα1</td><td></td></tr>
<tr><td>p-AMPK(172)</td><td>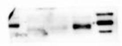</td></tr>
<tr><td>PRPS1</td><td></td></tr>
<tr><td>p-PRPS1(180)</td><td>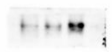</td></tr>
</table>

			β-actin	
Fig.8H	A875	IP	AMPKα1	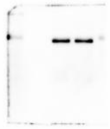
			p-AMPK(172)	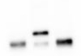
			PRPS1	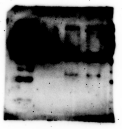
			p-PRPS1(180)	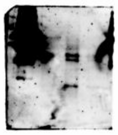
		Input	AMPKα1	
			p-AMPK(172)	
			PRPS1	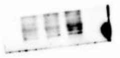

			p-PRPS1(180)	
			β-actin	
	SK-MEL-110	IP	AMPKα1	
			p-AMPK(172)	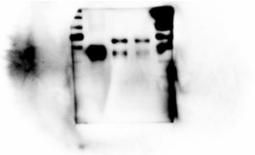
			PRPS1	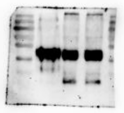
			p-PRPS1(180)	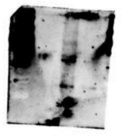
		Input	AMPKα1	
			p-AMPK(172)	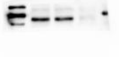

			PRPS1	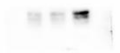
			p-PRPS1(180)	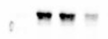
			β-actin	
Fig.9B	A875		NDUFS3	
			AMPK	
			p-AMPK(172)	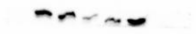
			PRPS1	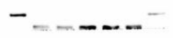
			p-PRPS1(180)	

		Tubulin	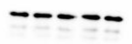
Fig.9C	SK-MEL-110	NDUFS3	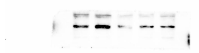
		AMPK	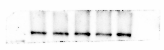
		p-AMPK(172)	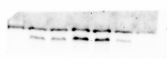
		PRPS1	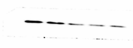
		p-PRPS1(180)	
		NDUFS3-OE-Tubulin	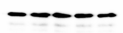
Fig.S2	A875	NDUFS3	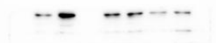

		β-actin	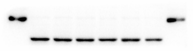
	SK-MEL-110	OE-NDUFS3	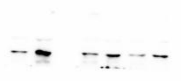
		KD-NDUFS3	
		NDUFS3-OE+KD-β-actin	
Fig.S3C	A875	NDUFS3-OE-Cyclin D1	
		NDUFS3-KD-Cyclin D1	
		NDUFS3-OE-CDK4	
		NDUFS3-KD-CDK4	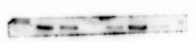

		NDUFS3-OE-P27	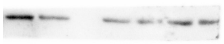
		NDUFS3-KD-P27	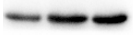
		Tublin	
	SK-MEL-110	NDUFS3-OE-Cyclin D1	
		NDUFS3-OE-CDK4	
		NDUFS3-KD-CDK4	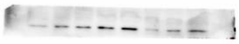
		NDUFS3-OE-P27	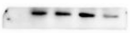
		NDUFS3-KD-P27	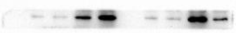

		Tublin	
